# Identification of Dynamic Active Sites Among Cu Species Derived from MOFs@CuPc for Electrocatalytic Nitrate Reduction Reaction to Ammonia

**DOI:** 10.1007/s40820-023-01091-9

**Published:** 2023-04-30

**Authors:** Xue-Yang Ji, Ke Sun, Zhi-Kun Liu, Xinghui Liu, Weikang Dong, Xintao Zuo, Ruiwen Shao, Jun Tao

**Affiliations:** 1 Key Laboratory of Cluster Science of Ministry of Education, School of Chemistry and Chemical Engineering, Liangxiang Campus, Beijing Institute of Technology, Beijing, 102488 People’s Republic of China; 2 Department of Chemistry, Sungkyunkwan University (SKKU), Suwon, 16419 Republic of Korea; 3 Department of Materials Physics, Saveetha School of Engineering, Saveetha Institute of Medical and Technical Sciences (SIMTS), Thandalam, Chennai, Tamilnadu 602105 India; 4 Beijing Advanced Innovation Center for Intelligent Robots and Systems and Institute of Engineering Medicine, Beijing Institute of Technology, Beijing, 100081 People’s Republic of China; 5 Key Laboratory of Bio-inspired Smart Interfacial Science and Technology of Ministry of Education, School of Chemistry, Beihang University, Beijing, 100191 People’s Republic of China

**Keywords:** Metal–organic frameworks, Copper phthalocyanine, Electrocatalytic nitrate reduction reaction

## Abstract

Direct electrochemical nitrate reduction reaction (NITRR) is a promising strategy to alleviate the unbalanced nitrogen cycle while achieving the electrosynthesis of ammonia. However, the restructuration of the high-activity Cu-based electrocatalysts in the NITRR process has hindered the identification of dynamical active sites and in-depth investigation of the catalytic mechanism. Herein, Cu species (single-atom, clusters, and nanoparticles) with tunable loading supported on N-doped TiO_2_/C are successfully manufactured with MOFs@CuPc precursors via the pre-anchor and post-pyrolysis strategy. Restructuration behavior among Cu species is co-dependent on the Cu loading and reaction potential, as evidenced by the advanced *operando* X-ray absorption spectroscopy, and there exists an incompletely reversible transformation of the restructured structure to the initial state. Notably, restructured CuN_4_&Cu_4_ deliver the high NH_3_ yield of 88.2 mmol h^−1^ g_cata_^−1^ and FE (~ 94.3%) at − 0.75 V, resulting from the optimal adsorption of NO_3_^−^ as well as the rapid conversion of *NH_2_OH to *NH_2_ intermediates originated from the modulation of charge distribution and *d*-band center for Cu site. This work not only uncovers CuN_4_&Cu_4_ have the promising NITRR but also identifies the dynamic Cu species active sites that play a critical role in the efficient electrocatalytic reduction in nitrate to ammonia.

**Supplementary Information:**

The online version contains supplementary material available at 10.1007/s40820-023-01091-9.

## Highlights

- Cu species with tunable loading supported on N-doped TiO_2_/C were successfully fabricated utilizing MOFs@CuPc precursors via the pre-anchor and post-pyrolysis strategy.
- Cu species with tunable loading supported on N-doped TiO_2_/C were successfully fabricated utilizing MOFs@CuPc precursors via the pre-anchor and post-pyrolysis strategy.
- Restructured CuN_4_&Cu_4_ performed the highest NH_3_ yield (88.2 mmol h^−1^ g_cata_^−1^) and FE (~94.3%) at − 0.75 V due to optimal adsorption of NO_3_^−^ and rapid conversion of the key intermediates.

**Supplementary Information:**

The online version contains supplementary material available at 10.1007/s40820-023-01091-9.

## Graphical abstract

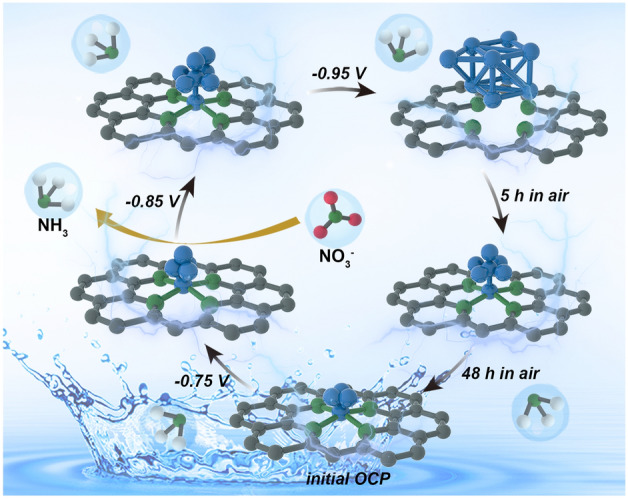

## Introduction

Ammonia (NH_3_) is an indispensable chemical used mainly in the manufacture of fertilizer, the synthesis of nitric acid and nitrate, and a refrigerant [1, 2]. The Haber–Bosch process, which utilizes N_2_ and H_2_ as input gases in a demanding environment (high temperature and pressure), is widely recognized as one of the greatest breakthroughs of the twentieth century and accounts for the overwhelming majority of yearly NH_3_ production [3, 4]. Compared to the Haber–Bosch process (high CO_2_ emissions and energy consumption), an environment-friendly NH_3_ synthesis by an electrocatalytic N_2_ reduction reaction (NRR) has garnered conspicuous attention, which enables the electrosynthesis of NH_3_ [5, 6]. Furthermore, nitrate (NO_3_^−^) is the usual contaminant in surface and ground water, exacerbating soil and water eutrophication and posing a threat to the environment and human health [7]. It could be an intriguing tactic for electrocatalytic NO_3_^−^ reduction reaction (NITRR) to NH_3_ under ambient circumstances via an eight-electron transfer reaction [8–10]. Hence, seeking low-cost, excellent selective, and efficient electrocatalysts capable of enabling both NH_3_ production and NO_3_^−^ conversion rate is critical.

Single-atom catalysts have been extensively recruited in various catalytic reactions due to their maximized atomic utilization and coordination diversity [11–13]. To date, metal–organic frameworks (MOFs) have been selected as the platform for the direct or indirect fabrication of single-atom catalysts on account of the multifarious anchoring mechanisms (e.g., spatial confinement, structural defects, and coordination strategies) in comparison with carbon nanotubes and graphene-based supported materials [14]. Among amine-functionalized MOFs, e.g., NH_2_-UiO66 Zr, NH_2_-MIL125 Ti (here denoted as aMIL), and NH_2_-MIL101 Al, the uncoordinated amine groups (–NH_2_) as the electron donors can stabilize and anchor single metal to synthesize single-atom electrocatalysts with well-defined metal-N_4_ structure [15–18].

In general, transition-metal atoms are susceptible to migrate and aggregate during pyrolysis with the high temperature-induced effect, forming metal nanoclusters and/or nanoparticles, which is always a thorny problem that seriously affects the targeted synthesis of highly loaded metal single-atom catalysts [19]. In addition, among the single-atom catalysts, Cu electrocatalysts can be restructured and induced by the special potential to form Cu nanoclusters and nanoparticles during electrocatalytic reduction reactions. In situ-formed metal nanoclusters or nanoparticles modulate the *d*-band center of the metal –N_4_ active site, optimize its electronic structure and improve catalytic performance [20, 21]. For instance, Fu and co-workers reported the transformation from Cu single-atom catalysts (CuN_4_ configuration) to the compound of Cu single-atom and nanoclusters during the alkaline oxygen reduction reaction (ORR). The restructured Cu-nanoclusters can regulate the *d*-band center of CuN_4_ and balance the free energy of intermediates, benefiting ORR activity [22]. Liu and co-workers found the reversible transformation of atomically dispersed Cu atoms and Cu_3_/Cu_4_ clusters during the alkalescent carbon dioxide reduction reaction (CO_2_RR) [23]. In applying NITRR, Wang and co-workers observed the dynamic reversible transformation between Cu single-atom catalysts (Cu loading: 1.0 wt%) and Cu_9_ nanoclusters with aggregation driven by the potential in an alkaline electrolyte and redispersion driven by oxidation at environmental conditions. Cao and co-workers verified in situ clustering of single-atom Cu in MOFs (Cu loading: 1.9 wt%) in Na_2_SO_4_ and NaNO_3_ electrolytes [24, 25]. In light of the aforementioned development, exploring the dual-driven behaviors of Cu species loading and reaction potential for electrocatalytic NH_3_ production is essential, especially for identifying the dynamical active sites.

Herein, Cu species (single-atom, clusters, and nanoparticles) anchored on N-doped TiO_2_/C (NTC) were synthesized via the pre-anchor and post-pyrolysis strategy. As a result, the restructuration behavior of Cu species was jointly related to Cu loading and reaction potential during electrocatalytic NITRR. Specifically, the higher copper loading and negative potential will more easily transform copper single atoms into copper clusters and particles. Cu1.5/NTC exhibited the highest NH_3_ yield with 88.2 mmol h^−1^ g_cata_^−1^ (0.044 mmol h^−1^ cm^−2^) and FE (~ 94.3%) at the potential of − 0.75 V versus RHE, which was superior to NTC, Cu0.7/NTC, and Cu3.2/NTC electrocatalysts. *Operando* XAS and density functional theory (DFT) calculation results confirmed that the restructured CuN_4_&Cu_4_ can enhance the adsorption ability of NO_3_^−^ and promote the rapid potential-determining step conversion of *NH_2_OH to *NH_2_ intermediates.

## Experimental Section

### Preparation of aMIL@CuPc-x Precursors

The synthesis of NH_2_-MIL125 (Ti) refers to our previous work with minor modifications [26]. In the synthesis of NH_2_-MIL125 (Ti)@CuPc precursor (aMIL@CuPc-x), NH_2_-BDC (840 mg, 4.6 mmol), C_12_H_28_O_4_Ti (0.90 mL, 3.3 mmol), and a series of amounts of C_32_H_16_CuN_8_ (CuPc) were successively added into the mixed solution containing anhydrous DMF and MeOH (60 mL, v/v = 9:1), and then stirred magnetically at room temperature for 1 h. Subsequently, the resulting solution was transferred to a Teflon-lined stainless steel autoclave and heated at 150 °C for 72 h until cooling to the room temperature. DMF and MeOH were alternately washed three times and centrifuged (8,000 rpm, 6 min), and then dried at 120 °C under dynamic vacuum for 24 h to obtain the final precursor powder. The additive amounts of CuPc were aMIL (0 mg), aMIL@CuPc-1 (46 mg, ~ 0.08 mmol), aMIL@CuPc-2 (92 mg, ~ 0.16 mmol) and aMIL@CuPc-3 (184 mg, ~ 0.32 mmol), respectively.

### Preparation of Derivatives

The powder precursors were calcined at 800 °C with the rate of 2 °C min^−1^ for 3 h in Ar. N-doped TiO_2_/C (NTC) with different Cu loading could be prepared by the precursors of pyrolysis aMIL@CuPc-x. ICP-OES showed that the Cu content was 0.673 (~ 0.7), 1.540 (~ 1.5) and 3.242 (~ 3.2) wt%, respectively. The samples were named in turn as NTC, Cu0.7/NTC, Cu1.5/NTC, and Cu3.2/NTC. The entire synthesis process is shown in Scheme S1.

### Electrochemical Measurements

All NITRR electrochemical measurements were performed on an electrochemical workstation (Ivium Vertex, Netherlands) in a three-electrode system using the H-type electrolytic cell separated by a Nafion 117 membrane (DuPont) at the room temperature (Fig. S1). Nafion 117 membrane was pretreated in the 5 wt% H_2_O_2_ solution at 80 °C for 1 h, rinsed by ultrapure water and then in ultrapure water at 80 °C for another 1 h, rinsed with ultrapure water several times. Typecially, as-prepared sample (5 mg) was dispersed in 900 μL of isopropanol (IPA) aqueous solution (v/v, 1:1) and 100 μL Nafion solution (5%), and ultrasonicated for 30 min. The homogeneous ink was dropped onto the commercial carbon paper (1 cm × 1 cm, mass loading: ca. 0.5 mg cm^–2^). Pt plate (1 cm × 1 cm), saturated calomel electrode (SCE) and above carbon paper were used as the counter, reference and working electrode in 0.5 M Na_2_SO_4_ and 50 ppm NaNO_3_ mixed solutions. The potentiostatic test was conducted at constant potentials for 2 h with a flow rate of Ar for 10 standard cubic centimeters per minute (sccm) and a stirring rate of 400 rpm. Cyclic voltammetry activation was not performed before the experiment, and the pH changes before and after the experiment were ignored. The potential reference equation (Eq. 1) was converted into the reversible hydrogen electrode (RHE).

1 $$E_{{{\text{RHE}}}} = E_{{{\text{SCE}}}} + 0.241{\text{ V}} + 0.0591 \times {\text{pH}}$$

### *Operando* XAS Experiment

The NITRR operando XAS experiment was tested in fluorescence mode at a BSRF 4B9A line station and used the home-built H-type cell referring to the NITRR experimental condition. Where the electrolyte was a mixed aqueous solution with Ar-saturated 0.5 M Na_2_SO_4_ and 50 ppm NaNO_3_, and the ink is uniformly dropped on a carbon paper of 1 cm × 2 cm (with a mass of about 2.5 mg cm^–2^), and the operando XAS experiment is started at a specific voltage without the cyclic voltammetry (CV) activation.

## Results and Discussion

### Characterization of MOFs@CuPc-x Precursors

**Fig. 1:**
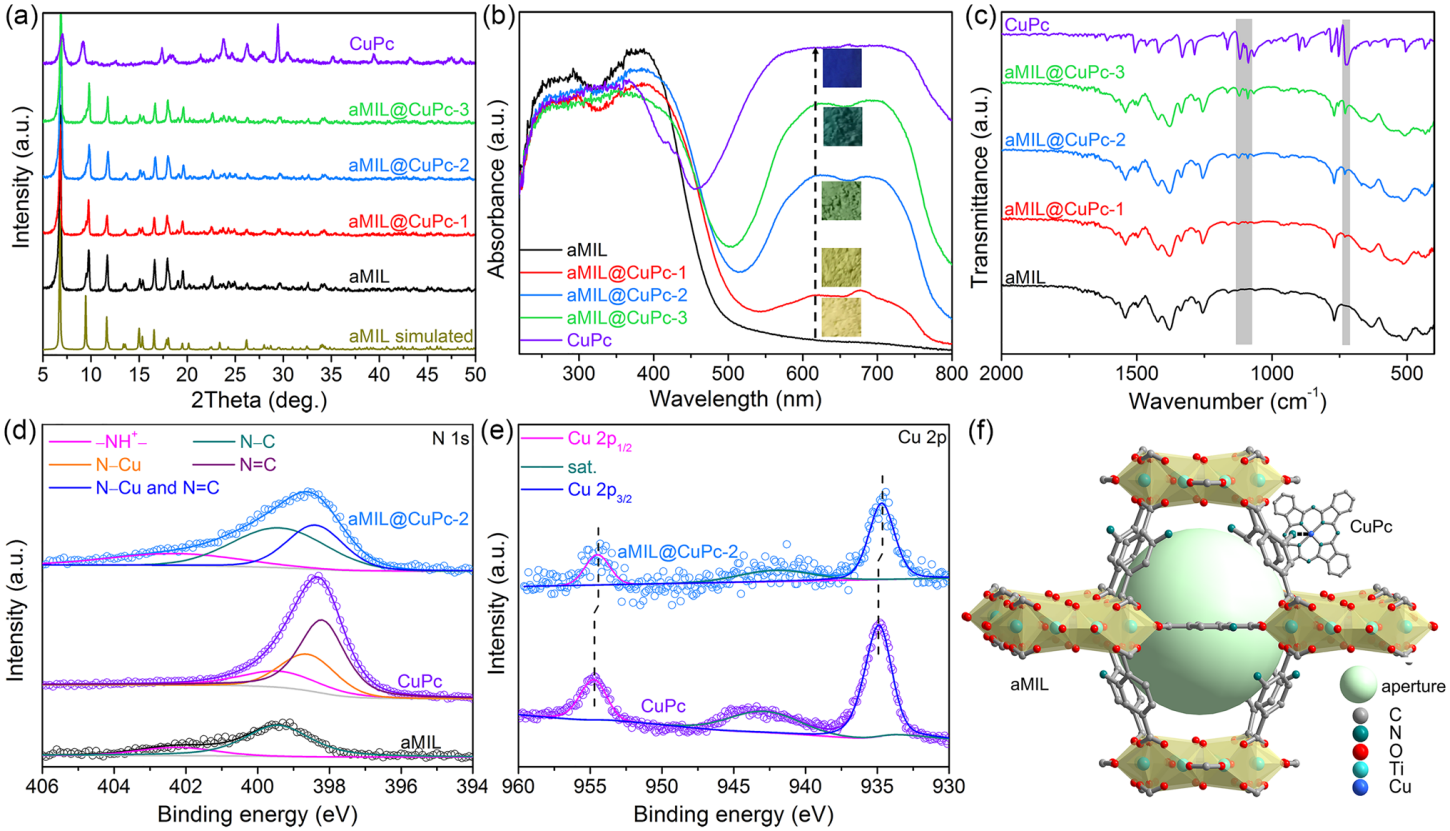
**a–c** PXRD patterns, UV–vis DRS and optical photograph, FT-IR spectra of aMIL, aMIL@CuPc-x, CuPc samples. **d, e** Typical high-resolution XPS spectra of N 1*s* and Cu 2*p* of aMIL, CuPc and aMIL@CuPc-2 samples. **f** Assembly mechanism between aMIL and CuPc

All of the experimental powder X-ray diffraction (PXRD) patterns of the as-synthesized aMIL@CuPc-based precursors (Fig. 1a) agree well with the simulated one of pure aMIL due to the similarity of the strong diffraction peaks between aMIL and CuPc or the lower mass loading of CuPc. Increased visible-region absorption and a gradual darkening of color with increasing CuPc loading are observable in the UV–vis diffraction spectra (DRS) and corresponding optical photographs (Fig. 1b) of aMIL, aMIL@CuPc-1, aMIL@CuPc-2, aMIL@CuPc-3, and CuPc samples, proving the presence of aMIL and CuPc composite. The vibrational peaks of aMIL@CuPc-based precursors in Fourier transform infrared (FT-IR) spectra (Fig. 1c) at approximately 1116, 1086, and 725 cm^−1^ correspond to C–H in-plane deformation (β_(C–H)_), C–N stretching in pyrrole (ν_(C–N)_), C–N out-of-plane deformation (γ_(C−N)_) except for the characteristic peaks of aMIL [27]. To uncover the link mode between aMIL and CuPc molecules, X-ray photoelectron spectroscopy (XPS) was characterized for aMIL, CuPc, and aMIL@CuPc-2 precursors. The presence of –NH–^+^ (402.3 eV) and N–C bonds (399.4 eV) among the –NH_2_ group in aMIL is confirmed by typical high-resolution XPS spectra of N 1*s* (Fig. 1d) [26]. In addition, there exist N–C bond (399.4 eV), pyrrolic N–Cu coordination form (398.6 eV), and N=C bond (398.2 eV) for the CuPc sample [28]. Due to the proximate binding energy of N–Cu and N=C bonds and higher half-peak breadth of N–C bonds given by both aMIL and CuPc, pyrrolic N–Cu and N=C bonds were joint fitted through deconvolution locating at 398.4 eV. Significantly, typical high-resolution XPS spectra of Cu 2*p* of aMIL@CuPc-2 slightly shift to the lower binding energy compared with that of CuPc in view of the electron-donation effect of –NH_2_ group [29], the axial N of –NH_2_ group over aMIL could be coordinated with CuPc to form [Ti_8_(OH)_4_O_8_(BDC-NH_2_)_6-x_]–(BDC-NH)_x_–(CuPc)_x_ (Fig. 1f). Compared to pure CuPc, the average coordination number (CN) of Cu–N bond over the aMIL@CuPc-2 increases from 3.72 to 4.20 (Fig. S4, Table S1). Meanwhile, aMIL@CuPc-based precursors retain the tetragonal plate shape of the aMIL matrix (Fig. S5), while CuPc molecules can form nanorod structures due to self-nucleation with higher CuPc loading [30].

### Structural Characterization of Cux/NTC Derivatives

**Fig. 2:**
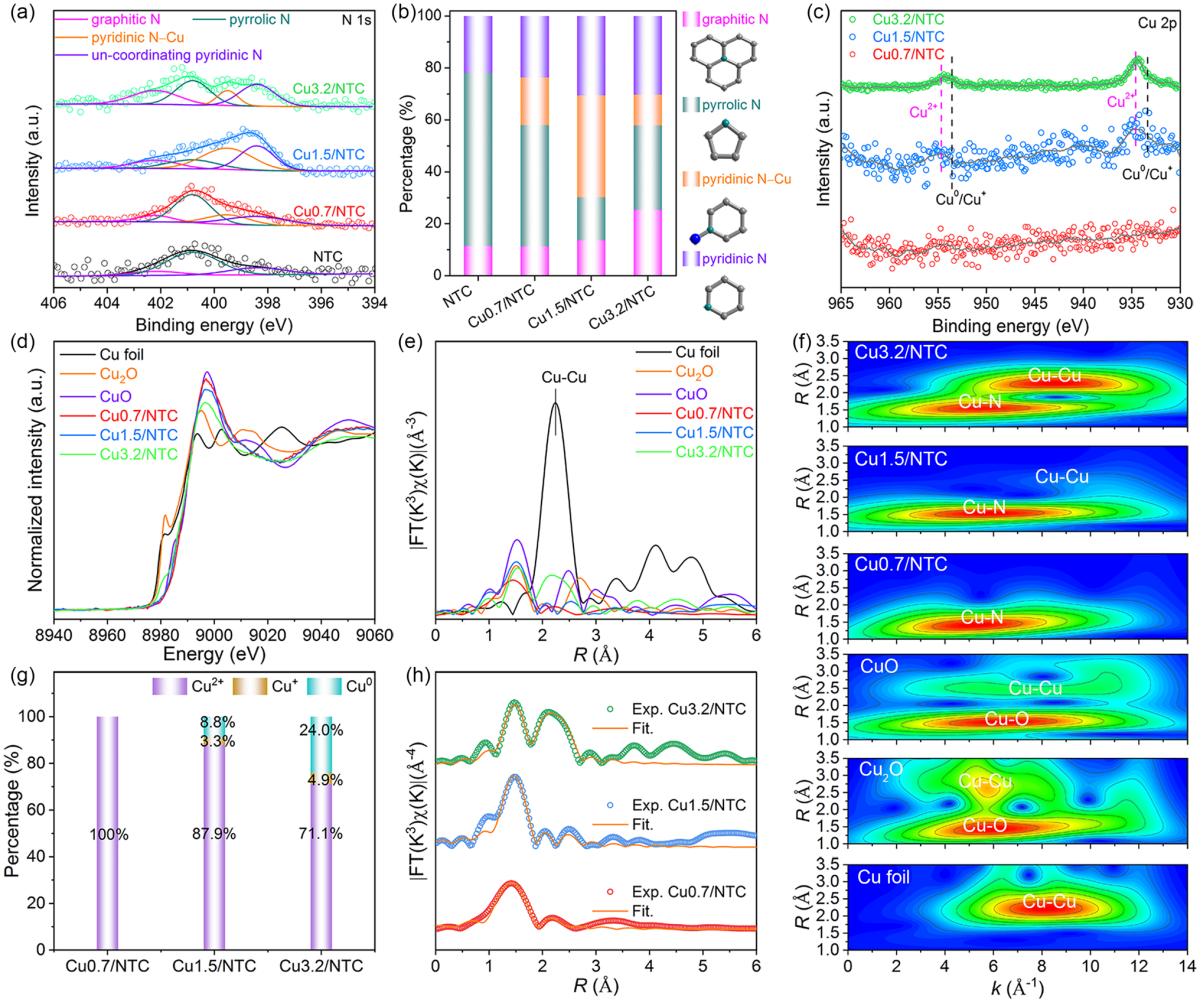
**a** High-resolution XPS of N 1*s*; **b** N contents and configurations; **c** Cu 2*p* for Cu0.7/NTC, Cu1.5/NTC, and Cu3.2/NTC. **D–f** Cu K-edge XANES, *k*^3^-weighted FT-EXAFS, and WT-EXAFS spectra of Cu foil, Cu_2_O, CuO, Cu0.7/NTC, Cu1.5/NTC, Cu3.2/NTC. **g, h** XANES LCF and FT-EXAFS spectra fitting curves of Cu0.7/NTC, Cu1.5/NTC, Cu3.2/NTC

The rational design of precursors with variable loading of CuPc affects the dispersity of Cu species among aMIL@CuPc-based derivatives to a certain extent. The PXRD patterns (Fig. S6, Table S2) substantiate the existence of the mixed crystal phase with Anatase and Rutile TiO_2_, and the mass ratio (*m*_Anatase_: *m*_Rutile_) is 2.34. Besides, a distinct diffraction peak exists at 43.3º of metallic Cu phase (JCPDS No: 04–0836) just for Cu3.2/NTC sample. Brunauer–Emmett–Teller (BET) specific surface area, pore volume, and Raman spectra (Fig. S7, Table S3) of NTC, Cu0.7/NTC, Cu1.5/NTC, and Cu3.2/NTC samples show that Cu1.5/NTC possess the biggest specific surface area, larger pore volume and the degree of graphitization, which can provide more active sites and enhance the conductivity of supported materials to accelerate the mass transfer of NITRR. The characteristic peaks of the high-resolution XPS N 1*s* spectra (Fig. 2a) at 402.2, 400.8, 399.5, and 398.4 eV correspond in turn to graphitized N, pyrrole N, pyridine N–Cu, and un-coordinating pyridine N, revealing the presence of N-doped C and Cu–N species [28]. Additionally, the content of pyrrole N–Cu of Cu3.2/NTC sample is lower than Cu0.7/NTC along with the progressive introduction of CuPc, indicating that Cu3.2/NTC contains a small amount of Cu–N species. High-resolution XPS of Cu 2p spectrum of Cu0.7/NTC (Fig. 2c) has a low signal-to-noise ratio, which can be attributed to the low Cu content. The peaks of Cu1.5/NTC at 955.0 and 934.7 eV can be described as Cu 2*p*_1/2_ and Cu 2*p*_3/2_ of Cu^2+^ [31], and the peaks of Cu3.2/NTC samples at 953.5 and 933.3 eV are Cu 2*p*1/2 and Cu 2*p*_3/2_ of Cu^0^ or Cu^+^ [32]. The high-resolution XPS of Cu 2*p* spectra for both Cu1.5/NTC and Cu3.2/NTC display a large half-peak width, indicating mixed valence states of Cu with 0, + 1, and + 2. Further pre-edge and white line peaks of the X-ray absorption near-edge structure (XANES) spectra at Cu K-edge (Fig. 2d) suggest that the average valence states of Cu successively are + 2 ≥ Cu0.7/NTC > Cu1.5/NTC > Cu3.2/NTC >  + 1. As shown in the *k*^3^-weighted Fourier-transformed extended X-ray absorption fine structure (FT-EXAFS) and wavelet transform extended X-ray absorption fine-structure (WT-EXAFS) spectra (Fig. 2d, f), Cu0.7/NTC, Cu1.5/NTC, and Cu3.2/NTC have the Cu–N scattering path in the first shell, while Cu1.5/NTC and Cu3.2/NTC sample show the Cu–Cu scattering path in the second shell compared with Cu foil, Cu_2_O, and CuO reference samples. The proportions of Cu^2+^, Cu^+^, and Cu^0^ for Cu1.5/NTC are 87.9%, 3.3% and 8.8%, the corresponding proportions for Cu3.2/NTC are 71.1%, 4.9% and 24.0% in the linear combination fitting (LCF) results of XANES spectra (Figs. 2g and S10), manifesting the generation of metallic Cu with the high temperature-induced effect during pyrolysis. The average CN of Cu–N bond (Figs. 2h and S11, Table S4) for Cu0.7/NTC, Cu1.5/NTC, and Cu3.2/NTC are 3.99 (~ 4), 4.02 (~ 4), and 3.61 (< 4), and the average *CN* of Cu–Cu bonds for Cu1.5/NTC and Cu3.2/NTC samples are 2.84 (~ 3) and 7.41 (~ 7) based on the proportion of Cu^0^ in XANES LCF.

### Morphology of Cux/NTC Derivatives

**Fig. 3:**
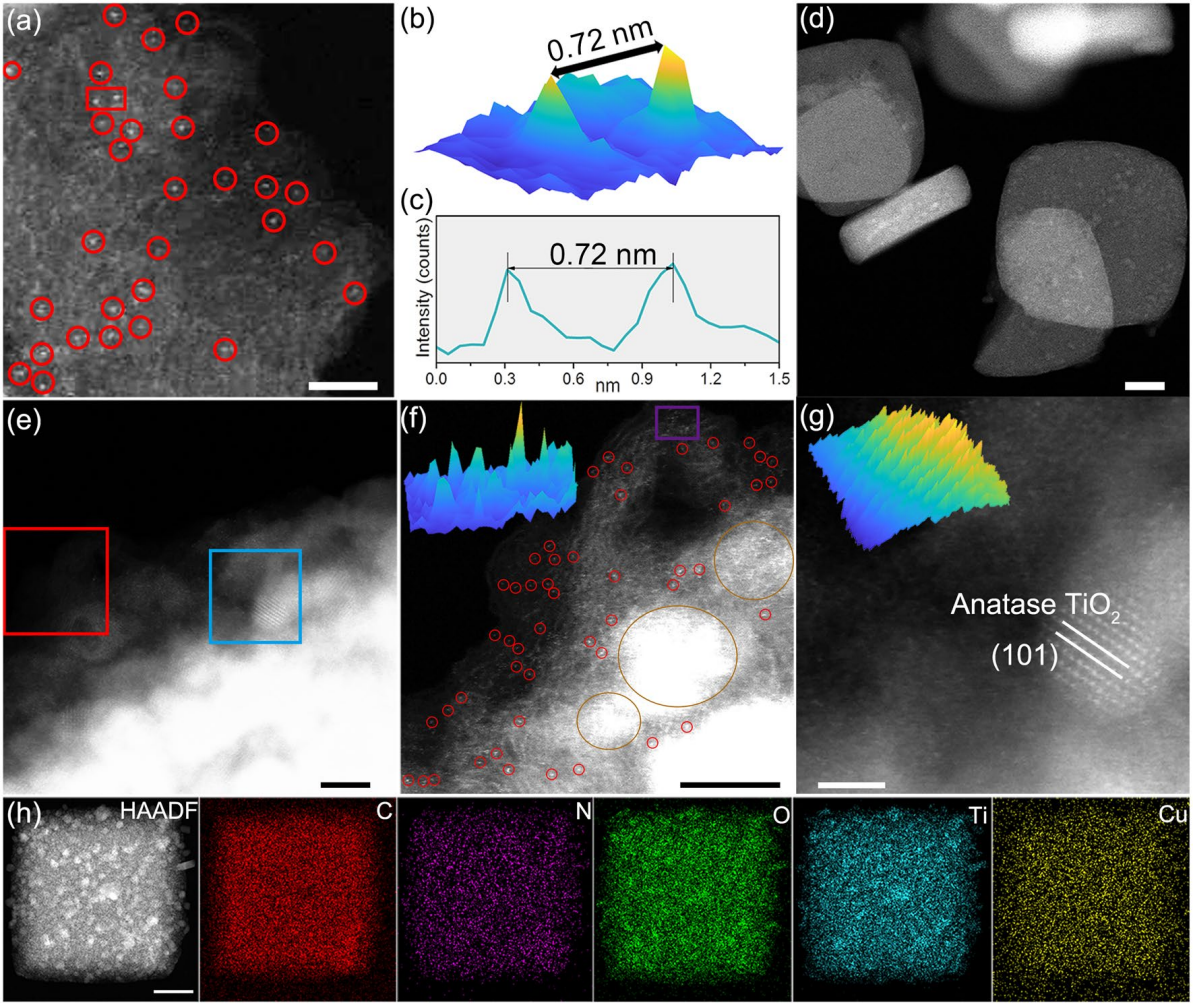
**a** AC HAADF-STEM image of Cu0.7/NTC. **b, c** 3D and line profile images of the red rectangular area in **a**. **d–g** HAADF-STEM images of Cu1.5/NTC, and **f, g** are the enlarged images of the red and blue rectangular areas in **e**. **h** EDX mapping images of Cu1.5/NTC. Scale bar: **a** 2 nm, **e–g** 5 nm, **d, h** 200 nm

The dispersion state of Cu species and supported materials among Cu0.7/NTC, Cu1.5/NTC, and Cu3.2/NTC were observed by aberration-correction high-angle-annular-dark-field scanning transmission electron microscopy (AC HAADF-STEM) and high-resolution transmission electron microscopy (HRTEM). AC HAADF-STEM image (Fig. 3a–c) of Cu0.7/NTC reveals that Cu is monodispersed on the NTC and the distance of neighbored Cu atoms is relatively large (~ 0.72 nm). AC HAADF-STEM images of Cu1.5/NTC (Fig. 3d–f) provide vital evidence for the coexistence form of Cu species with both monodispersity and clusters (small red cycles and big dark yellow circles in Fig. 3f). Further, Fig. 3g (the enlarged image of the blue rectangular area of Fig. 3e) and HRTEM images of Cu1.5/NTC (Fig. S13) uncover the existence of both anatase and rutile TiO_2_ on the surface of N-doped C supported materials, which further confirmed with the well-defined elements distribution of C, N, O, Ti, and Cu (Fig. 3h). Furthermore, Cu nanoparticles with exposed (111) crystal plane load on the surface of NTC material for Cu3.2/NTC sample, integrating with the EDX mapping images (Fig. S14).

### Electrocatalytic NITRR Performances

**Fig. 4:**
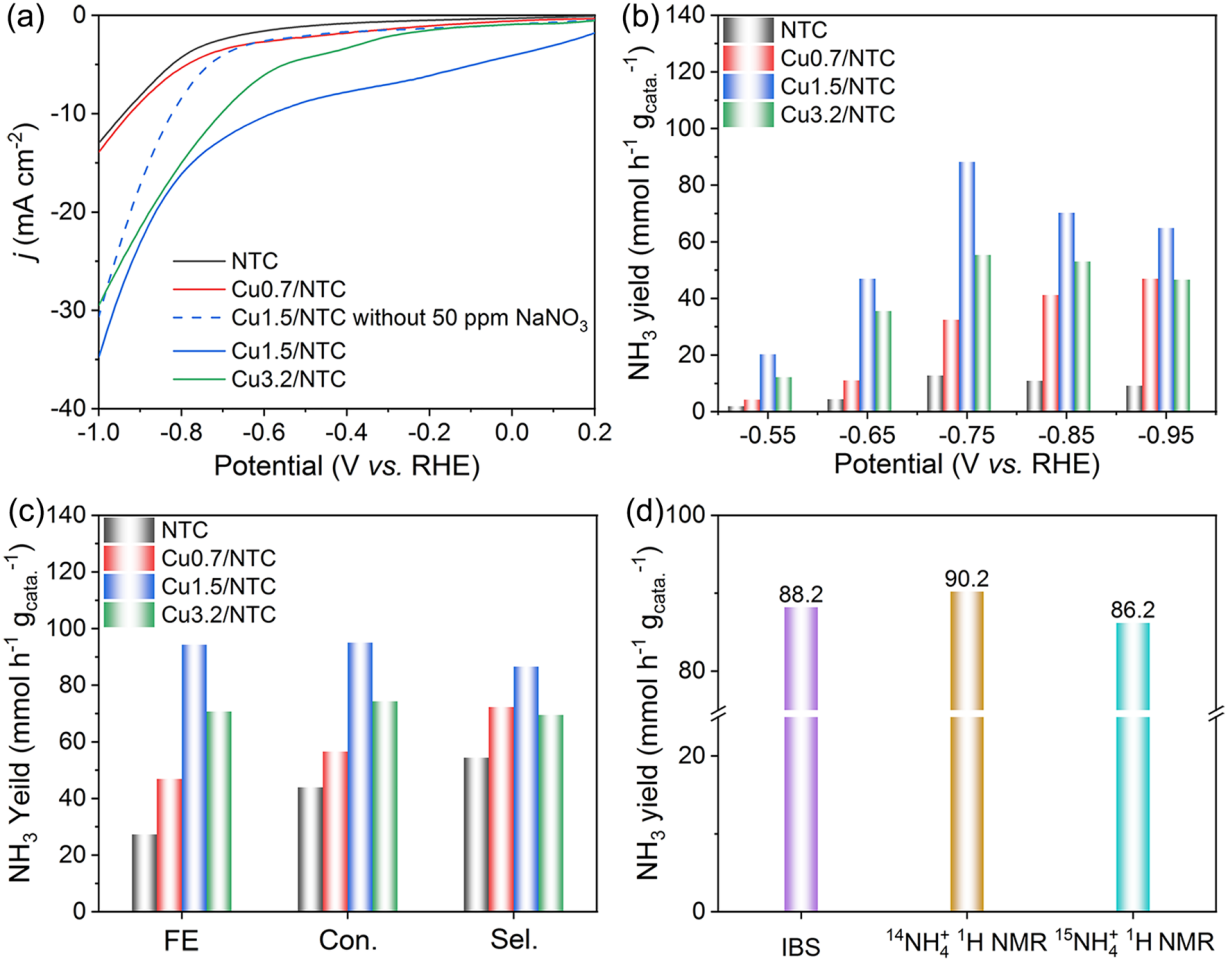
**a** LSV polarization curves of NTC, Cu0.7/NTC, Cu1.5/NTC and Cu3.2/NTC in 0.5 M Na_2_SO_4_ electrolytes with 50 ppm NaNO_3_, and without 50 ppm NaNO_3_ for Cu1.5/NTC. **b** NH_3_ yield of NTC, Cu0.7/NTC, Cu1.5/NTC and Cu3.2/NTC. **c** The comparison of FE, conversion rate (Con.) and selectivity (Sel.) of NTC, Cu0.7/NTC, Cu1.5/NTC and Cu3.2/NTC samples at –0.75 V. **d** Comparison of NH_3_ yield via IBS and ^1^H NMR methods

The NITRR performances of NTC, Cu0.7/NTC, Cu1.5/NTC, and Cu3.2/NTC electrocatalysts were evaluated in 0.5 M Na_2_SO_4_ and 50 ppm NaNO_3_ electrolytes. As exhibited in the linear sweep voltammetry (LSV) polarization curves (Fig. 4a), the current density (*j*) of Cu0.7/NTC, Cu1.5/NTC, and Cu3.2/NTC are higher than that of NTC, and Cu1.5/NTC has the highest current density in the range of 0.2 to − 1.0 V, indicating that the introduction of appropriate Cu species can promote the NITRR process. Compared to the LSV curves of Cu1.5/NTC before and after adding 50 ppm NaNO_3_, the potential range from − 0.55 to − 0.95 V versus RHE was selected to quantify the NH_3_ production performance. After the potentiostatic experiment at the potentials of − 0.55,  − 0.65,  − 0.75,  − 0.85, and − 0.95 V for 2 h, the products of NH_3_, NO_2_^−^ and catalysis substrate (NO_3_^−^) were detected via colorimetric methods (Figs. S15–S17). Cu1.5/NTC delivers best NH_3_ yield (88.2 mmol h^−1^ g_cata_^−1^) and lower NO_2_^−^ yield at − 0.75 V, which is superior to NTC, Cu0.7/NTC and Cu3.2/NTC at this potential (Figs. 4b and S18). Meanwhile, Cu1.5/NTC presents the optimal FE (94.3%), 95.0% conversion rate, and 86.6% selectivity of NO_3_^−^ to NH_3_ (Fig. 4c), which confirms that Cu is the main active site of NITRR, agreeing well with that the introduction of appropriate Cu species significantly increased the *A*_ECSA_ to enhance the performance of electrocatalytic NH_3_ production (Fig. S19). Meanwhile, NH_3_ yield and FE for Cu1.5/NTC show no significant decay during the continuous long-term tests at − 0.75 V (Fig. S20). To ensure the N source and accurately estimate NH_3_ yield, multi-position in one mode of ^15^N isotope labeling experiment, indophenol blue spectrophotometric (IBS), and ^1^H nuclear magnetic resonance (NMR) methods were engaged by conducting the product from the potentiostatic experiment for Cu1.5/NTC at − 0.75 V using blank Na_2_SO_4_ solution, Na^14^NO_3_ and Na^15^NO_3_ as N sources (Figs. S21–S23). Based on the prominent characteristic triple and double peaks of ^14^NH_4_^+^ and ^15^NH_4_^+^ (Figs. S22 and S23) [33], NH_4_^+^ yield quantified with ^1^H NMR method is very close to the results of IBS method (Fig. 4d). The reliable NH_3_ yield and FE of Cu1.5/NTC executed in neutral electrolyte are on par with the majority of published Cu-based electrocatalysts (Table S5).

### Restructuration Process Detected with *Operando* XAS

**Fig. 5:**
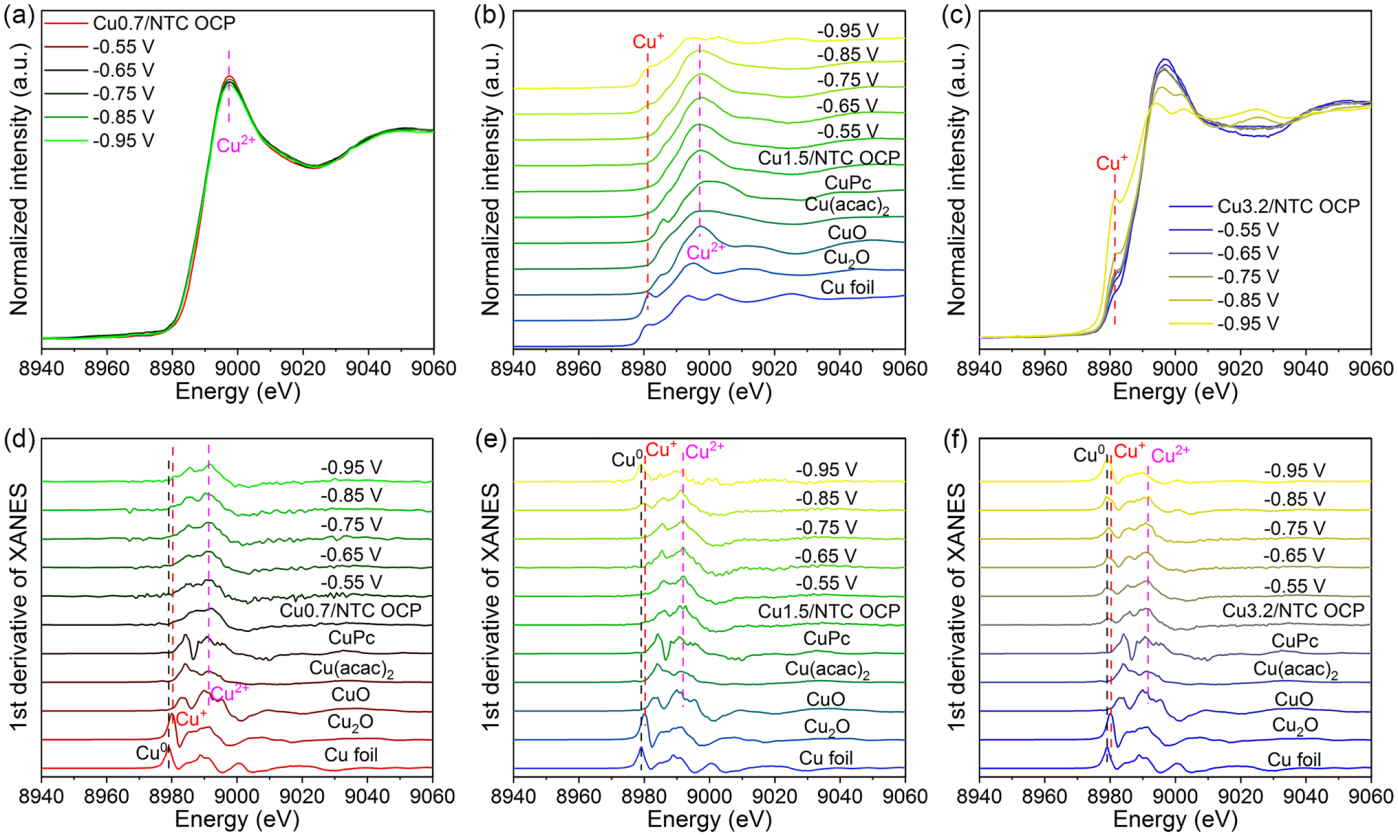
**a–c** *Operando* XANES spectra of Cu0.7/NTC, Cu1.5/NTC, and Cu3.2/NTC. **d-f** 1st derivative of XANES spectra of Cu0.7/NTC, Cu1.5/NTC and Cu3.2/NTC

*Operando* XAS experiment is frequently employed to accurately record the dynamic structural changes of metal activity sites during electrocatalysis reactions of single-atom catalysts [34, 35]. *Operando* XAS experiments of Cu0.7/NTC, Cu1.5/NTC, and Cu3.2/NTC were conducted at open circuit potential (OCP),  − 0.55, − 0.65,  − 0.75,  − 0.85, and − 0.95 V versus RHE for Cu K-edge using the home-built H-type cell referring to the NITRR experimental condition, and Cu foil, Cu_2_O, CuO, Cu(acac)_2_, CuPc are reference samples. There are no obvious differences in the structure and valence state at different potentials in the XANES and 1st derivative of XANES spectra for Cu0.7/NTC electrocatalyst (Fig. 5a, d). The characteristic peaks in the XANES spectra of Cu1.5/NTC (Fig. 5b) at 8988.7 eV are attributed to the electron transition from Cu^2+^ 1*s* to Cu^2+^ 4*p*, while the peaks of − 0.85 and − 0.95 V located at ~ 8982 eV originate the electron transition from Cu^+^ 1* s* to Cu^+^ 4*p*_y_ [36], which indicate that Cu1.5/NTC electrocatalyst begins undergoing the obvious transition from Cu^2+^ to Cu^+^ at − 0.85 V. The 1st derivative of XANES spectra (Fig. 5e) at the potentials of − 0.85 and − 0.95 V have a big half-peak width at ~ 8979 and 8980 eV [37], revealing that Cu1.5/NTC has the mixed valence states of Cu^0^, Cu^+^, and Cu^2+^ at these potentials. The characteristic peaks of Cu^+^ in the XANES spectra and Cu^0^ in the 1st derivative of XANES spectra for Cu3.2/NTC at all the potentials (Fig. 5c, f) become more robust with the negative shift of reaction potential, suggesting that the Cu species among Cu3.2/NTC experience the significant reduction process of Cu^2+^  → Cu^+^  → Cu^0^.

To understand the valence state and coordination environment of Cu species thoroughly for Cu0.7/NTC, Cu1.5/NTC, and Cu3.2/NTC electrocatalysts, we pay more attention to resolving the FT-EXAFS, XANES LCF, and WT-EXAFS spectra at the representative potentials of OCP,  − 0.75,  − 0.85, and − 0.95 V. The *k*^3^-weighted FT-EXAFS fitted spectra together with the fitting results of XANES LCF spectra (Figs. S24 and S25, Table S6) show that Cu0.7/NTC still keeps the dominant configuration of Cu–N_4_ at OCP,  − 0.75,  − 0.85,  − 0.95 V. However, the partial Cu^+^–N_x_ generated at the − 0.75 V potential could detach from the anchorage of N-doped C among Cu– N_4_ and undergo the significant restructuration driven by the potential at − 0.85 V to form Cu_2_ clusters with Cu–N_4_ (CuN_4_&Cu_2_), and then further restructuring to form CuN_4_&Cu_6_ at − 0.95 V. The weak Cu–Cu bond (k: 8.9 Å^−1^) and apparent Cu–Cu bonds (k: 9.9 Å^−1^) could be detected in the WT-EXAFS spectra of − 0.85 and − 0.95 V for Cu0.7/NTC (Fig. S26), except the findable Cu–N bonds at the potentials from OCP to − 0.75 V. Discriminatively, the CN of Cu–N bonds at OCP, − 0.75, − 0.85,  − 0.95 V for Cu3.2/NTC are 3.58, 2.89, 1.55, 0.41 in succession, showing that the primary configurations of Cu species at OCP and − 0.75 V are Cu^2+^ − N_4_, while Cu^0^ nanoparticles (NPs) exist at − 0.85 and − 0.95 V (Figs. S27–S29, Table S7). The restructuration behavior in Cu3.2/NTC system can be jointly dependent on the Cu loading amount and reaction potential since Cu^0^ content of Cu3.2/NTC grows substantially more than that of Cu0.7/NTC at the same potentials.

**Fig. 6:**
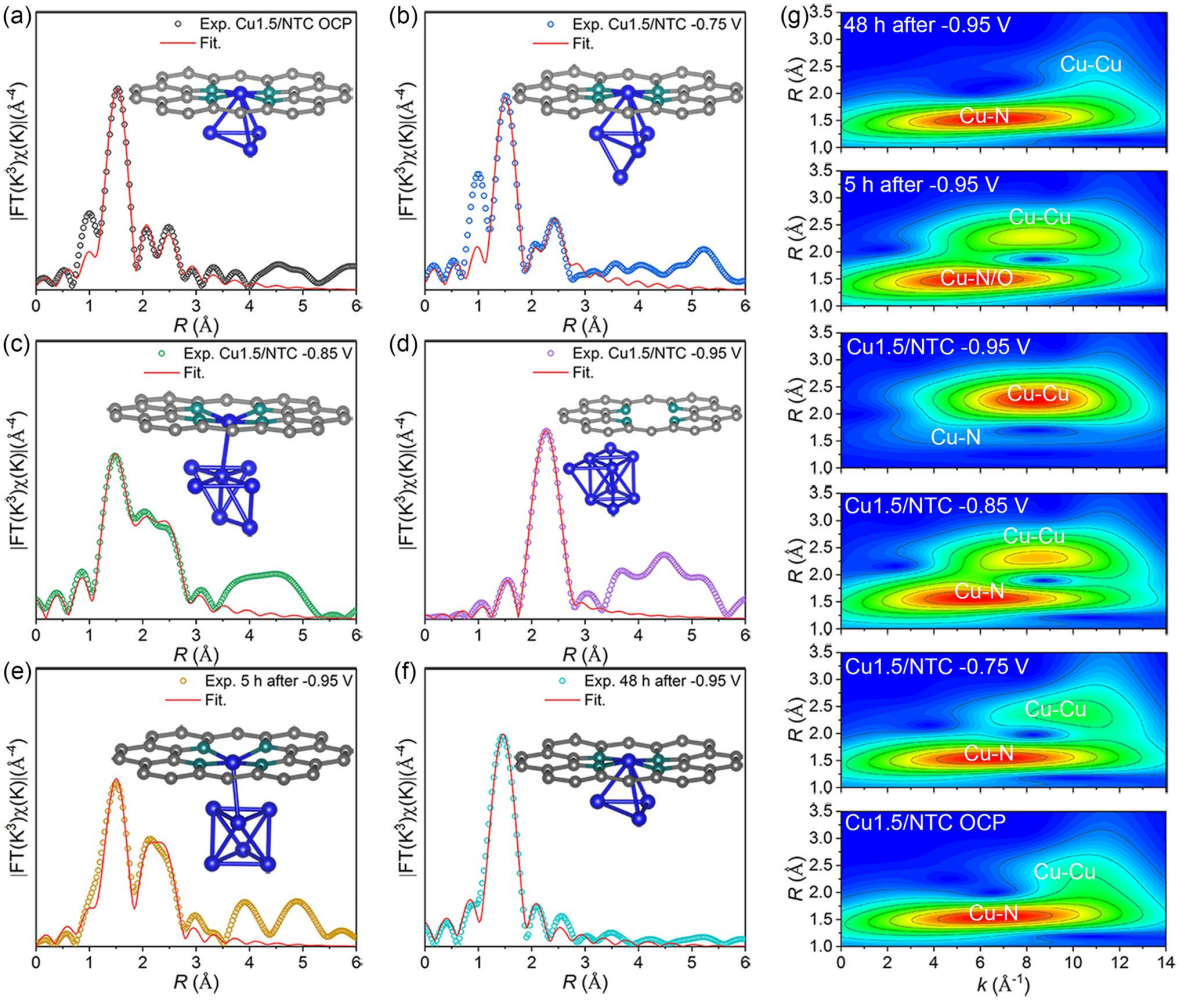
**a–f** The fitting spectra of R space for Cu1.5/NTC at OCP, − 0.75, − 0.85, − 0.95 V, 5 and 48 h after − 0.75 V in air, and the corresponding structures in the inset. **g** The WT-EXAFS spectra. Gray, green and blue balls, respectively, represent C, N, and Cu atoms

Given the *k*^3^-weighted FT-EXAFS fitting spectra integrated with the XANES LCF results of Cu1.5/NTC (Figs. 6a–d, S30–S32, and Table S8), the CN of Cu–N bonds at OCP, − 0.75, − 0.85, − 0.95 V are 3.90, 3.87, 3.58, 0.59, and the CN of Cu–Cu bonds are 2.90, 4.12, 7.29, and 8.85, so it is reasonable to assume that Cu1.5/NTC had CuN_4_&Cu_3_, CuN_4_&Cu_4_, CuN_4_&Cu_7_, and Cu_9_ structures at the aforementioned potentials (inset of Fig. 6a–d). Intrigued by the stability of restructuring Cu clusters, *ex-situ* XANES, 1st derivative of XANES, R space, FT-EXAFS fitting spectra, WT-EXAFS, and XANES LCF spectra detected 5 and 48 h after − 0.95 V in the air (Figs. 6e–g, S30–S33) clarify the transformation of valence states from metallic Cu to the oxidation state of Cu, accompanied by the incompletely reversible transformation from Cu_9_ clusters to CuN_4_&Cu_3_ (initial OCP). Moreover, the bond length in the first shell of exposure in the air for 5 h after − 0.95 V (1.96 Å) is marginally bigger than that of exposure in the air for 48 h (1.94 Å), which can result from the bond between Cu and light atoms (N/O) during the oxidation process of metallic Cu, following by the formation of [Cu(OH)_4_]^2−^ intermediate on the wet carbon paper of an alkaline solution. Ultimately, intermediates could be anchored again on the –N_4_C_x_ sites among the supported materials due to the chelating capacity of Cu^2+^ [24, 35].

**Fig. 7:**
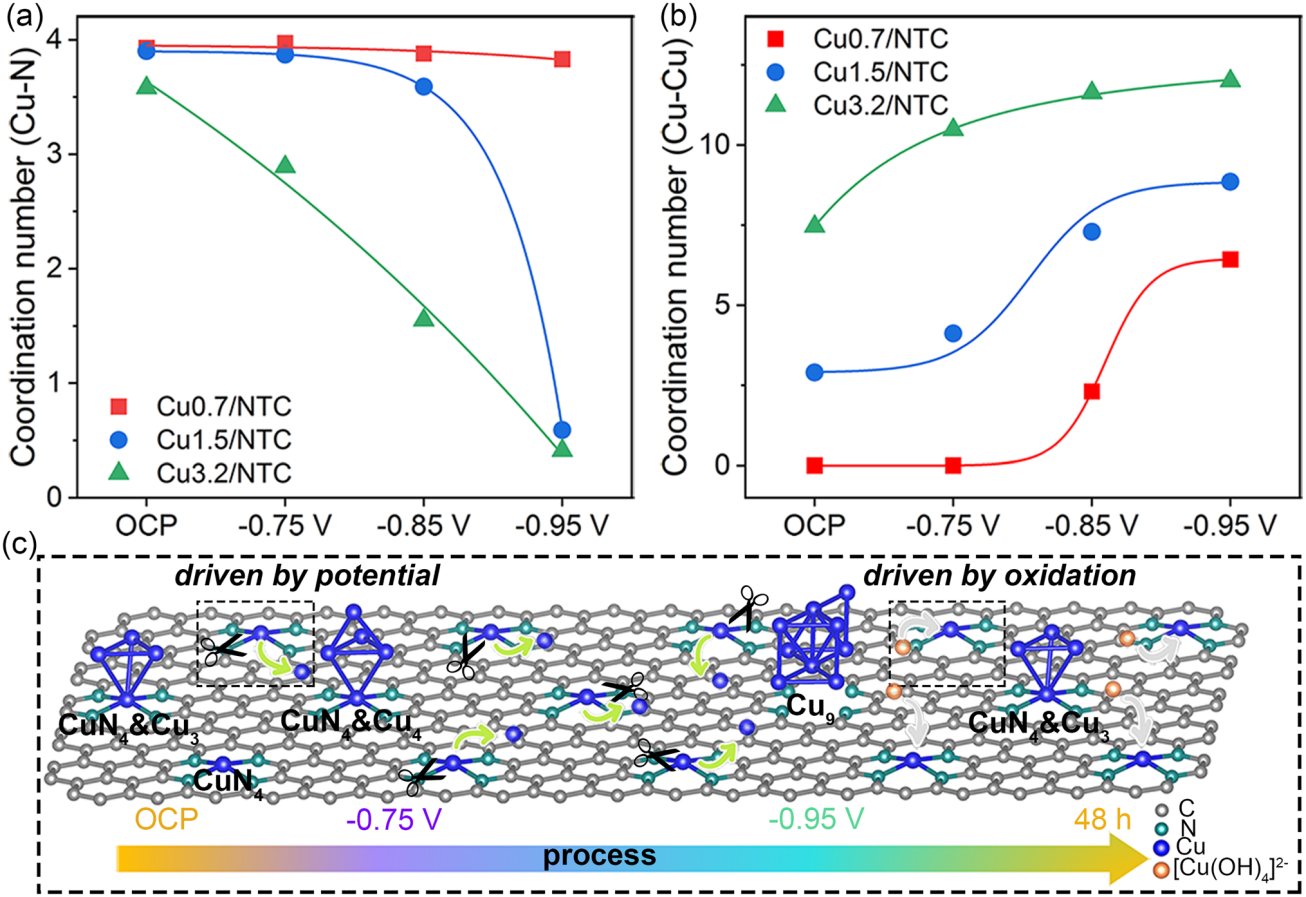
**a, b** Nonlinear fitting curves of the typical reaction potential versus the *CN* (Cu–N and Cu–Cu bonds) among Cu0.7/NTC, Cu1.5/NTC, and Cu3.2/NTC electrocatalysts. **c** The possible electrochemically driven remodeling and oxidation-driven redispersion mechanisms for Cu1.5/NTC

The relationships among the CN, Cu loading, and reaction potential were further investigated using the nonlinear fitting curves of the typical reaction potential versus the *CN* (Cu–N and Cu–Cu bonds) toward Cu0.7/NTC, Cu1.5/NTC, and Cu3.2/NTC electrocatalysts (Fig. 7a, b). Potential intervals of the dominant Cu–N_4_ configuration in Cu0.7/NTC, Cu1.5/NTC, and Cu3.2/NTC electrocatalysts are OCP to − 0.95 V, OCP to − 0.85 V, OCP to − 0.75 V, while the *CN* maximum increments of Cu–Cu bonds occur at − 0.95, − 0.85, − 0.75 V, indicating that the potentials of electrochemical restructuration are shifted positively with the increased loading of Cu. Consequently, the restructuration behavior among Cu species in this system co-depends on the Cu loading and reaction potential. Furthermore, the possible mechanisms of aggregation driven by potential and redispersion driven by oxidation integrated with chelation for Cu1.5/NTC are described in Fig. 7c and further verified with the EDX mapping of Cu1.5/NTC exposed in the air after − 0.95 V (Fig. S34).

### Electrocatalytic NITRR Mechanisms

**Fig. 8:**
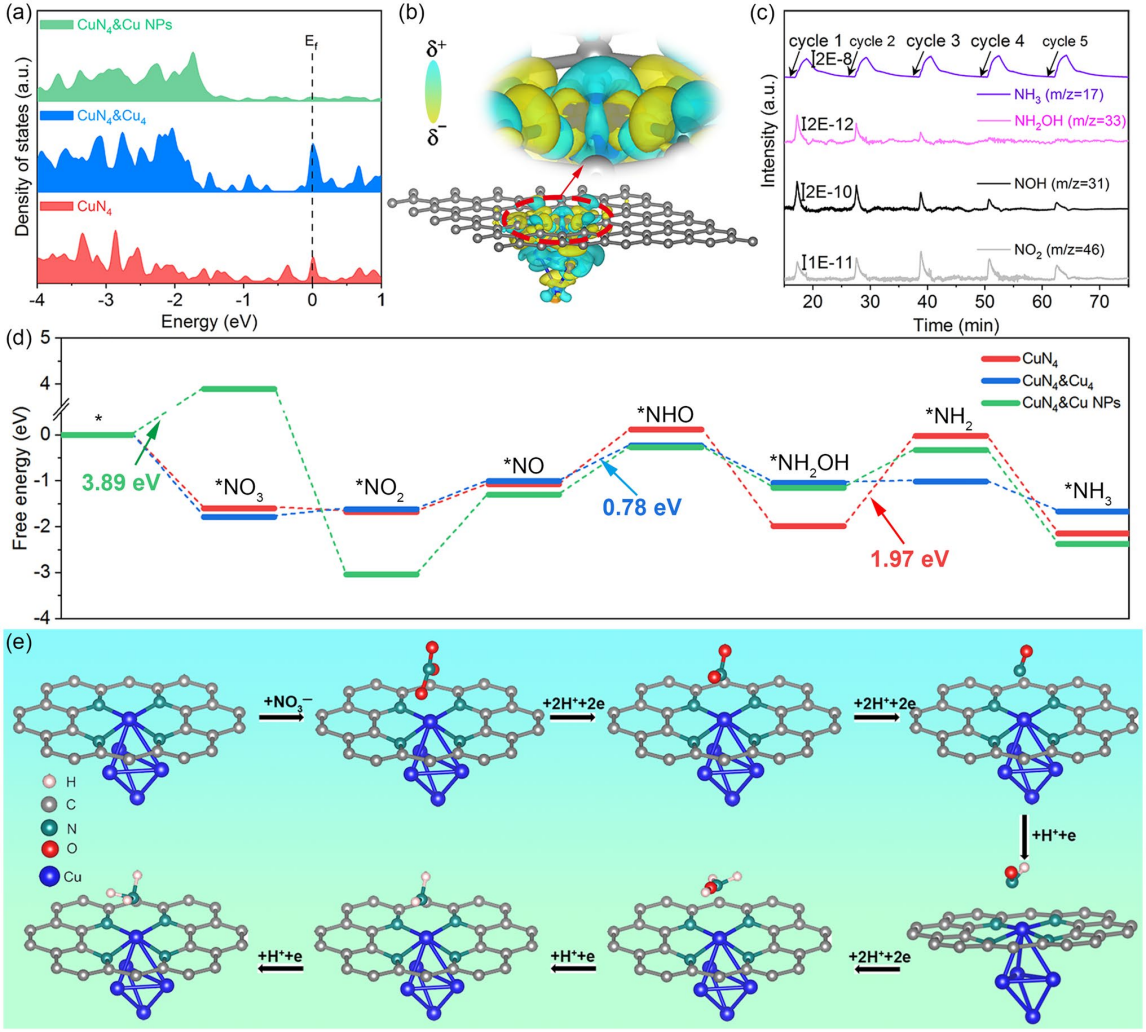
**a** PDOS CuN_4_, CuN_4_&Cu_4,_ and CuN_4_&Cu NPs. **b** EDD distribution of CuN_4_&Cu_4_ model. The yellow and cyan regions represent electron accumulation and depletion. **c** Online DEMS measured at –0.75 V for NITRR over Cu1.5/NTC. **d** The free energy paths of NH_3_ production for CuN_4_, CuN_4_&Cu_4_ and CuN_4_&Cu NPs. **e** The minimum energy pathway of NH_3_ production for CuN_4_&Cu_4_ configuration. Color code: H (light pink), C (gray), N (green), O (red), Cu (blue). (Color figure online)

To further evaluate the origin of the NITRR catalytic activity, density functional theory (DFT) calculations were executed with the multi-component models (Fig. S35) of Cu species, including Cu single-atom (CuN_4_), Cu single-atom and clusters (CuN_4_&Cu_4_), Cu single-atom and nanoparticles with (111) facet (CuN_4_&Cu NPs), based on the fitting structures of FT-EXAFS and the variation trend of mass activity (Fig. S36) [38, 39]. The *d*-band center of projected density of states (PDOS) for CuN_4_&Cu_4_ model (*d*_center_ =  − 2.56 eV) is closest to the Fermi level than that of CuN_4_ and CuN_4_&Cu NPs (Figs. 8a and S37), signifying that the strong interaction between CuN_4_ and Cu clusters upshifted the *d*-band center to improve the adsorption ability of reactants and intermediates [40, 41]. Charge density difference (EDD) distributions of CuN_4_, CuN_4_&Cu_4_, and CuN_4_&Cu NPs were calculated (Figs. 8b and S38). As a result, introducing the Cu cluster to CuN_4_ (CuN_4_&Cu_4_) can ameliorate the charge distribution of the Cu single-site and modulate the electric structure with high activity for NITRR compared with CuN_4_ and CuN_4_&Cu NPs, crucial for invigorating the sluggish NITRR kinetics [42]. Online differential electrochemical mass spectrometry (DEMS) offers robust technical support for capturing volatile intermediates and products [43]. Additionally, mass signals were acquired during the chronoamperometry test at − 0.75 V for five cycles (Fig. 8c), indicating mass signals of *m*/*z* = 17, 31, 33, and 46 are assigned to the intermediate products or the molecular fragments of NH_3_, NOH, NH_2_OH, and NO_2_. According to the results of online DEMS, and integrated with the deoxygenation process of NO_3_^−^ (*NO_3_ → *NO_2_ → *NO) and hydrogenation process of *NO intermediate [44], we calculated the free energy pathway of NH_3_ formation for each step in detail upon the constructed models with the most stable adsorption configurations, especially for the first step of hydrogenation site of *NO intermediate. Notably, the first hydrogenation site of the *NO intermediate for CuN_4_, CuN_4_&Cu_4_, and CuN_4_&Cu NPs preferentially forms *NHO rather than *ONH (Fig. S39), which coincides with the DEMS results with the by-products (NOH and NH_2_OH). At the initiation step of the possible NITRR pathway on CuN_4_, CuN_4_&Cu_4_, and CuN_4_&Cu NPs utilized Perdew–Burke–Ernzerhof (PBE) functional, the adsorption of reactant NO_3_^–^ is a spontaneous behavior toward CuN_4_ and CuN_4_&Cu_4_ (more favorable), while the NO_3_^–^ adsorption on CuN_4_&Cu NPs with the free energy changes (ΔG) of 3.89 eV is thermodynamically unfavorable (Figs. 8d, e, and S40-S41. Tables S9 and S10) [41, 45]. Besides, the potential-determining steps (PDS) of CuN_4_ model is *NH_2_OH to *NH_2_ (ΔG = 1.97 eV), which is apparently higher than CuN_4_&Cu_4_ model (ΔG = 0.03 eV) despite the PDS of CuN_4_&Cu_4_ model is *NH to *NOH (ΔG = 0.78 eV), indicating Cu cluster can optimize reaction energy barrier of *NH_2_OH to *NH_2_. Meanwhile, the results of solvent model and the local density approximation (LDA) functional were adopted in NITRR calculations, which gave similar tendency in CuN_4_&Cu_4_ configuration to PBE data (Figs. S43-S44). In a nutshell, the synergistic coupling between the CuN_4_ and Cu clusters modulates the charge distribution and the electric structure with the suitable *d*-band center, enhancing the adsorption ability of NO_3_^−^ and accelerating the rapid conversion from *NH_2_OH to *NH_2_ simultaneously.

## Conclusions

In conclusion, we have exhaustively investigated the restructuration behavior in the electrocatalytic NITRR process by employing Cu species with tunable loading supported on N-doped TiO_2_/C at potentials ranging from OCP to − 0.95 V versus RHE based on the *operando* XAS. Electrochemical restructuration is the reduction process of Cu with a positive valence state to metallic Cu, and then Cu^0^ can migrate and aggregate to form Cu clusters or nanoparticles. The restructuration behavior of Cu species is jointly dependent on Cu loading and reaction potential, i.e., the higher Cu loading and the more negative potential, the easier restructuration of Cu species to form Cu clusters or nanoparticles with higher nucleation numbers. *Operando* XAS and electrocatalytic NITRR performances perform that Cu1.5/NTC with CuN_4_&Cu_4_ configuration could deliver the highest NH_3_ yield with 88.2 mmol h^−1^ g_cata_^−1^ (0.044 mmol h^−1^ cm^−2^) and FE (~ 94.3%) at − 0.75 V, which is superior to NTC, Cu0.7/NTC (CuN_4_ configuration), and Cu3.2/NTC (CuN_4_&Cu_10_ configuration). DFT calculation results reveal that Cu clusters with appropriate nuclear numbers modulate the charge distribution and the *d*-band center of Cu single-site, thus promoting the adsorption ability of NO_3_^−^ and accelerating the conversion of the key intermediates (*NH_2_OH → *NH_2_). This work sheds light on the restructuring behavior trigger, allowing for more informed decisions in the rational design of highly active NITRR electrocatalysts.

### Supplementary Information

Below is the link to the electronic supplementary material.

Supplementary file1 (PDF 5059 KB)
