# Supplementary material for: Identification of Dynamic Active Sites Among Cu Species Derived from MOFs@CuPc for Electrocatalytic Nitrate Reduction Reaction to Ammonia
Source: Nanomicro Lett. 2023 Apr 30;15:110. doi: 10.1007/s40820-023-01091-9 (PMC10149566; doi:10.1007/s40820-023-01091-9)
Supplement: Supplementary file 1 — Supplementary file1 (PDF 5059 KB) [file 40820_2023_1091_MOESM1_ESM.pdf]

Supporting Information for

Identification of Dynamic Active Sites among Cu Species Derived from MOFs@CuPc for Electrocatalytic Nitrate Reduction Reaction to Ammonia

Xue-Yang Ji[1], Ke Sun[1], Zhi-Kun Liu[1], Xinghui Liu[2, 3, *], Weikang Dong[4], Xintao Zuo[5], Ruiwen Shao[4], Jun Tao[1, *]

[1]Key Laboratory of Cluster Science of Ministry of Education, School of Chemistry and Chemical Engineering, Liangxiang Campus, Beijing Institute of Technology, Beijing 102488, People's Republic of China

[2]Department of Chemistry, Sungkyunkwan University (SKKU), Suwon 16419, Republic of Korea

[3]Department of Materials Physics, Saveetha School of Engineering, Saveetha Institute of Medical and Technical Sciences (SIMTS), Thandalam, Chennai 602105, Tamilnadu, India

[4]Beijing Advanced Innovation Center for Intelligent Robots and Systems and Institute of Engineering Medicine, Beijing Institute of Technology, Beijing 100081, People's Republic of China

[5]Key Laboratory of Bio-inspired Smart Interfacial Science and Technology of Ministry of Education, School of Chemistry, Beihang University, Beijing 100191, People's Republic of China

*Corresponding authors. E-mail: taojun@bit.edu.cn (Jun Tao), liuxinghui119@gmail.com (Xinghui Liu)

S1 Experimental Section

S1.1 Reagents

All the chemicals were received from commercial resources and directly utilized without further purification. 2-Aminoterephthalic acid (NH_2-BDC, ≥ 99%) was purchased from Alfa Aesar (USA). Titanium tetraisopropanolate ($C_{12}H_{28}O_4Ti$, 99%), N, N-dimethylformamide (DMF, ≥ 99.5%), anhydrous N, N-dimethylformamide (99.8%, water ≤ 50 ppm), methanol (MeOH, ≥ 99.5%), anhydrous methanol (99.9%, water ≤ 50 ppm), sodium hydroxide (NaOH, ≥ 98%), sulfanilamide ($C_6H_8N_2O_2S$, 99%), N-(1-Naphthyl)ethylenediamine dihydrochloride ($C_{12}H_{16}Cl_2N_2$, 99%), sodium nitrite ($NaNO_2$, 99.99%), phosphoric acid (H_3PO_4, 85wt. % in water), sulfamic acid (NH_3SO_3, 99%), deuterium oxide (D_2O, 99.9%), dimethyl sulfoxide-d_6 (C_2D_6OS, 99.8%), sodium sulfate (Na_2SO_4, ≥ 99%), maleic acid ($C_4H_4O_4$, 99%), 2-propanol (C_3H_8O, 99.7%) were purchased from Admas Chemical Reagent Co. Ltd. Sodium nitrate ($NaNO_3$, 99.99%), ^{15}N-labeled sodium nitrate ($Na^{15}NO_3$, ≥ 98.5%), ammonium chloride (NH_4Cl, 99.5%), ^{15}N-labeled ammonium chloride (NH_4Cl, ≥ 98%), salicylic acid ($C_7H_6O_3$, ≥ 99%), sodium citrate dehydrate ($C_6H_5Na_3O_7$•$2H_2O$, 99%), sodium nitroprusside ($C_5FeN_6Na_2O$, 99%), copper(I) oxide (Cu_2O, 99%), copper(II) oxide (CuO, 99%), β-copper(II) phthalocyanine ($C_{32}H_{16}CuN_8$, > 90%) were purchased from Aladdin Chemical Reagent Co. Ltd. Sodium hypochlorite solution (NaClO, 0.1 M) was purchased from Macklin Chemical Reagent Co. Ltd. Ultrapure water (Millipore, 18.2 MΩ·cm) was used throughout all the experiments.

S1.2 General Characterizations

Powder X-ray diffraction (PXRD) patterns of the obtained samples were acquired on a Shimadzu XRD-6000 diffractometer with Cu Kα radiation (λ = 1.54056 Å), the scan rate was set as 5 ° min^{-1} under a step of 0.02°. Rietveld refinements were performed using the GSAS program with the EXPGUI interface [S1, S2], and the PXRD data were collected with a 1 ° min^{-1} rate from 5° to 120°. Fourier transform infrared (FT-IR) measurements were performed on a Thermo IS5 FT-IR spectrometer with KBr pellets. Thermogravimetric analysis-differential thermal analysis (TGA-DTA) measurements were performed on a HITACHI STA300 from room temperature to 400 °C with a heating rate of 5 °C min^{-1} in air. Raman spectra were recorded on a micro-Raman spectrometer consisting of a sample chamber coupled with an RH controller, a gaseous precursor generator, an optical microscope (DMLM; Leica) for observing droplet morphology, and a confocal Raman spectrometer (inVia; Renishaw) with a 514.5 nm argon-ion laser (model LS-514; Laser Physics) as the excitation source with a power of 30 mW. The morphologies of samples were determined with scanning electron microscopy (SEM) and EDS line scan (ZEISS-EVO18, 10 kV), transmission electron microscopy (TEM) and EDX (Talos F200X, ThermoFisher, 300 kV), and aberration-corrected high-angle annular dark-field scanning transmission electron microscopy (HAADF-STEM) at an accelerating voltage of 200 kV (JEM-ARM300F, JEOL). Inductively coupled plasma optical emission spectrometer (ICP-OES) was performed using the Agilent 5800 and dissolved with aqua regia in advance. N_2 adsorption-desorption isotherms were degassed at 150 °C for 12 h and subsequently measured at 77 K on a nitrogen adsorption-desorption instrument (Kubo-X1000, China). UV-Vis DRS were recorded by a UV-2600 (Shimadzu) spectrophotometer with quantified as-prepared MOFs-based precursors using $BaSO_4$ as a reference in the wavelength from 200 to 800 nm. X-ray photoelectron spectroscopy (XPS) measurements were performed on a Thermo Fisher Scientific ESCALAB 250Xi system with Al Kα radiation (photoelectron energy: 1486.6 eV), and the C1s peak at 284.5 eV was used to calibrate the peak positions. ^{1}H nuclear magnetic resonance (NMR) spectra were monitored on an Avance III HD 700 MHz spectrometer (Bruker Avance, German).

S1.3 XAS Measurements

X-ray absorption spectra (XAS) were recorded in fluorescence mode at the beamline 4B9A station of the Beijing Synchrotron Radiation Facility (BSRF). The measured data were processed with ATHENA, HAMA and ARTMIS IFEFFIT software packages to obtain X-ray absorption near-edge structure (XANES), Fourier transform extended X-ray absorption fine structure (FT-EXAFS) and wavelet transform extended X-ray absorption fine structure (FT-EXAFS) spectra. Cu and Ti K-edge k^3-weighted EXAFS spectra were obtained by normalizing with the edge-jump step, and k^3-weighted χ(k) data at the K edge were Fourier transformed to R space using hanning windows (dk = 1.0 $Å^{-1}$) to separate the EXAFS contributions from different coordination shells and obtain the coordination informations based on Eq. (S1) [S3]. During the calculation of coordination number for Cu cluster or nanoparticle, the Cu^0 proportion obtained by the Linear Combination Fitting (LCF) of XANES spectra was calculated according to Eq. (S2) [S4]. The XANES spectra of Cu foil, Cu_2O, and Cu OCP were selected as the standard spectra of Cu^0, Cu^+, and Cu^{2+}.

$$\chi(k) = \sum_j \frac{N_j S_0^2 F_j(k)}{k R_j^2} exp\,[-2k^2\sigma_j^2] exp\,[\frac{-2R_j}{\lambda(k)}] sin\,[2kR_j + \emptyset_j(k)] \qquad \text{(S1)}$$

$$CN^* = \frac{CN_{Cu\text{-}Cu}}{P_{Cu^0}} \qquad \text{(S2)}$$

S1.4 Online Differential Electrochemical Mass Spectrometry

Online differential electrochemical mass spectrometry (DEMS) was measured on QAS 100 (Linglu Instruments Co., Ltd.) referring to the electrocatalytic NITRR conditions. The

potentiostatic test was performed at –0.75 V *vs.* RHE, and five cycles were performed when the baseline remained stable to minimize the error of the experiment.

S1.5 Calculation of Electrochemical Active Surface Area (ECSA)

The electrochemical active surface area is determined by CV curves based on the electrochemical double-layer capacitance (C_{dl}) in a potential window nearly non-Faradaic process with different scan rates of 10, 20, 40, 60, 80, and 100 mV s^{-1}. C_{dl} was calculated according the slope of the scan rates vs. geometric current density, and ECSA was obtained with the following Eq. (S3):

$$A_{ECSA} = C_{dl}/(C_s \text{ per } cm^2_{ECSA}) \quad \text{(S3)}$$

C_s is the specific capacitance for a flat surface at the range of 20 to 60 μF cm^{-2}, which is frequently assumed to be 40 μF cm^{-2}.

S1.6 Determination of Ammonia

Indiphenol blue method was used to measure the generated NH_3 [S5]. The concentration-absorbance curves were calibrated using a series of concentrations of NH_4Cl solution, and the well-linear relation fitting curve (y = 0.5613x – 0.0013, R^2 = 0.9992) was obtained by three times independent calibrations. Typically, electrolyte was taken from the cathodic chamber and diluted to 2 mL, and then 2 mL of 1 M NaOH solution containing 5 wt% $C_7H_6O_3$ and 5 wt% $C_6H_5Na_3O_7$, 1 mL of 0.05 M NaClO and 0.2 mL of 1 wt% $C_5FeN_6Na_2O$ were added to the above solution. After standing at room temperature for 2 h, the UV-Vis absorption spectra were measured at a wavelength of 655 nm.

S1.7 Determination of Nitrite

The NO_2^- in liquid products were detected with the Griess method [S6]. The following ingredients were added to ultrapure water (25 mL) and stirred to form a clear solution: $C_6H_8N_2O_2S$ (1.0 g), $C_{12}H_{16}Cl_2N_2$ (0.1 g), and H_3PO_4 (5 mL), which was employed as the color reagent. The concentration-absorbance curve was calibrated by using a series of standard $NaNO_2$ solutions (y = 3.1967x + 0.0048, R^2 = 0.9999). In detail, a certain quantity of electrolyte obtained from the electrolytic cell was diluted to a 5 mL solution, and then the color reagent (0.1 mL) was subsequently added to the above-mentioned solution and mixed uniformly. After allowing the solution to settle for 10 min, the absorption intensity (540 nm) was measured.

S1.8 Determination of Nitrate

NO_3^- was determined by mixing 0.1 mL of 1 M HCl and 0.01 mL of 0.8 wt% sulfamic acid solution with 5 mL electrolyte diluent, and then the aforementioned solution was measured the absorption intensity at a wavelength of 220 nm (A_{220}) and 275 nm (A_{275}) [S7]. The final absorbance value (A) was evaluated using this equation: $A = A_{220} - 2\times A_{275}$, and different concentrations of $NaNO_3$ solution to calculate the well-linear relation fitting curve (y = 0.2031x + 0.0045, R^2 = 0.9997).

S1.9 $Na^{15}NO_3$ Isotope-labeling Experiment

$Na^{15}NO_3$ was selected as the feeding N-source to verify the source of produced ammonia. In the experiment of NITRR, 30 mL electrolyte containing 0.5 M Na_2SO_4 and 50 ppm $Na^{15}NO_3$–^{15}N were added into the cathode cell, and the final NH_3 electrolyte were assessed with 700 MHz ^1H NMR.

S1.10 NMR Determination of Ammonia

The produced $^{14}NH_3$ or $^{15}NH_3$ was also detected with 700 MHz ^1H NMR [S8]. The concentration-integral area ($NH_4Cl/C_4H_4O_4$) curves were calibrated with a series of NH_4Cl solution concentrations using 0.04 wt% maleic acid ($C_4H_4O_4$) in DMSO-d_6 as the internal

standard, and the well-linear relation fitting curve was obtained. Electrolyte (0.5 mL) was taken from the cathodic chamber, the pH of the electrolyte was adjusted to ~2 using 0.5 M H_2SO_4 with a certain volume, and then 0.1 mL 0.04 wt% $C_4H_4O_4$ in DMSO-d_6 was added to the above solution.

S1.11 Calculation of NITRR Performance

Faradaic efficiency of ammonia:

$$FE_{NH_3} = 8 \times F \times C_{NH_3} \times V/(M_{NH_3} \times Q) \tag{S4}$$

Yield rate of ammonia:

$$Y_{NH_3} = C_{NH_3} \times V/(M_{NH_3} \times t \times S) \tag{S5}$$

$$Y_{NH_3} = C_{NH_3} \times V/(M_{NH_3} \times t \times m_{cat.}) \tag{S6}$$

Faradaic efficiency of nitrite:

$$FE_{NO_2^-} = 2 \times F \times C_{NO_2^-} \times V/(M_{NO_2^-} \times Q) \tag{S7}$$

Yield rate of nitrite:

$$Y_{NO_2^-} = C_{NO_2^-} \times V/(M_{NO_2^-} \times t \times S) \tag{S8}$$

Conversion rate:

$$Con. = \Delta C_{NO_3^-}/C_0 \times 100\% \tag{S9}$$

The selectivity of ammonia:

$$S_{NH_3} = C_{NH_3}/\Delta C_{NO_3^-} \times 100\% \tag{S10}$$

Where F is the Faradaic constant (96485 C mol^{-1}), C is the mass concentration, V is the volume of electrolyte (30 mL), t is reaction time (1 h), Q is the total charge traveling through the electrode ($Q = \int_0^t j\mathrm{d}t$, j representes the geometric current density), S is the geometric area of carbon paper under the electrolyte (1 cm^2).

S1.12 Density Functional Theory Computational Details

All calculations were implemented using the Vienna Abinitio Simulation Package (VASP) code based on Density Functional Theory (DFT) [S9, S10]. For the following calculations of properties, General gradient approximation (GGA) was used with the Perdew–Burke–Ernzerhof (PBE) functional to describe the exchange-correlation potential [S11]. All structural models were entirely relaxed until the ionic Hellmann–Feynman forces were smaller than 0.001 eV/Å, the energy tolerances were less than 10^{-6} eV/atom. The interaction between core electrons and valence electrons was described using the frozen-core projector-augmented wave (PAW) method. Wave functions were expanded in a plane wave basis with high energy using plane-wave cutoff energy of 500 eV, and the corresponding gamma-centered Monkhorst-Pack electronic wavevector *k*-point samplings were denser than 0.2 $Å^{-1}$ [S12]. Meanwhile, the implicit solvation calculation was performed using VASPsol [S13, S14], a software package that incorporates solvation into VASP within a self-consistent continuum model. Note that the aqueous solution was adopted by use the dielectric constant $\varepsilon_b = 80.0$, width of dielectric cavity $\sigma = 0.6$, the cutoff charge density $\rho cut = 0.0025$ $Å^{-3}$ and a surface tension parameter of 0.525 meV/$Å^2$. The default parameters for VASPsol are used unless otherwise indicated in the text. To verify the reliability of our calculation results, the local density approximation (LDA) function was adopted for describing the exchange-correlation potential [S15]. For Gibbs free energy *(ΔG)* of the nitride reduction is defined as below:

$$\Delta G = \Delta E_{DTF} + \Delta E_{ZPE} - T\Delta S \tag{S11}$$

Where ΔE_{DFT} is the DFT electronic energy difference of each step, and T is the temperature (T = 300 K). ΔE_{ZPE} and ΔS are the difference in zero-point energy and entropy change, respectively.

common path:

$$NO_3^- + * \rightarrow *NO_3 \quad (S12)$$

$$NO_3* + 2H^+ + 2e^- \rightarrow *NO_2 + H_2O \quad (S13)$$

$$*NO_2 + 2H^+ + 2e^- \rightarrow *NO + H_2O \quad (S14)$$

*ONH path:

$$*NO + H^+ + e^- \rightarrow *ONH \quad (S15)$$

*NHO path:

$$*NO + H^+ + e^- \rightarrow *NHO \quad (S16)$$

$$*NHO + 2H^+ + e^- \rightarrow *NH_2OH \quad (S17)$$

$$*NH_2OH + H^+ + e^- \rightarrow *NH_2 + H_2O \quad (S18)$$

$$*NH_2 + H^+ + e^- \rightarrow *NH_3 \quad (S19)$$

S2 Supplementary Scheme

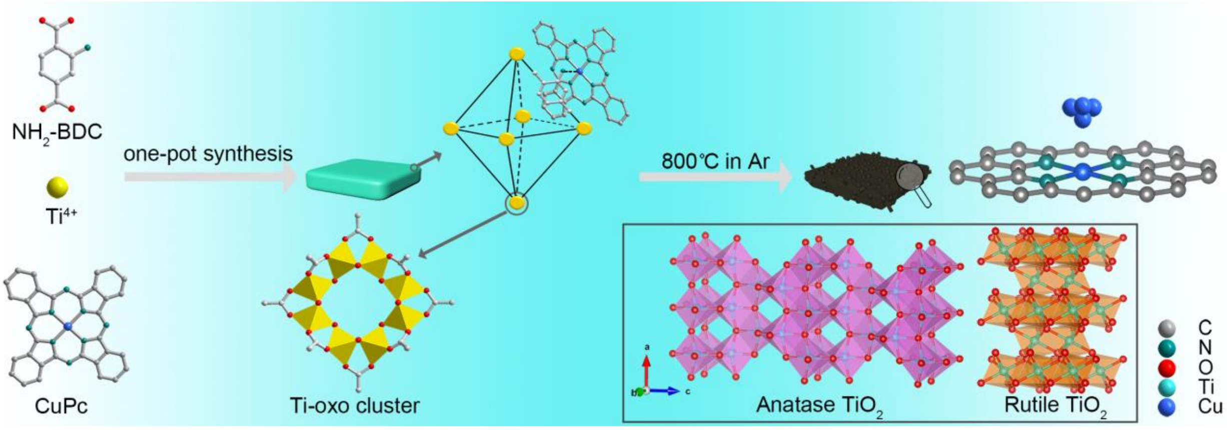

Scheme S1 Illustration showing the growth process of Cu_x/NTC

3 Supplementary Figures

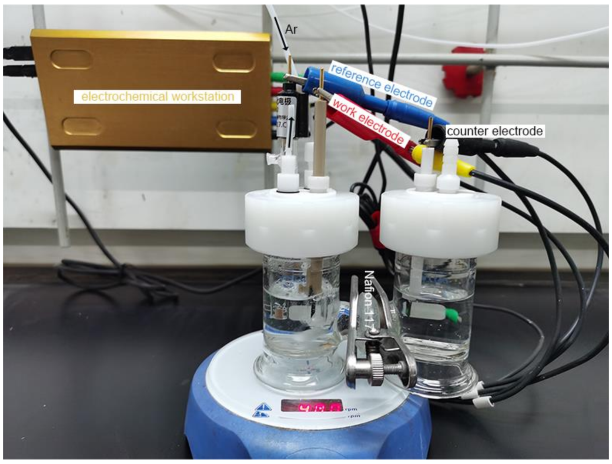

Fig. S1 The photograph of electrocatalytic NITRR process

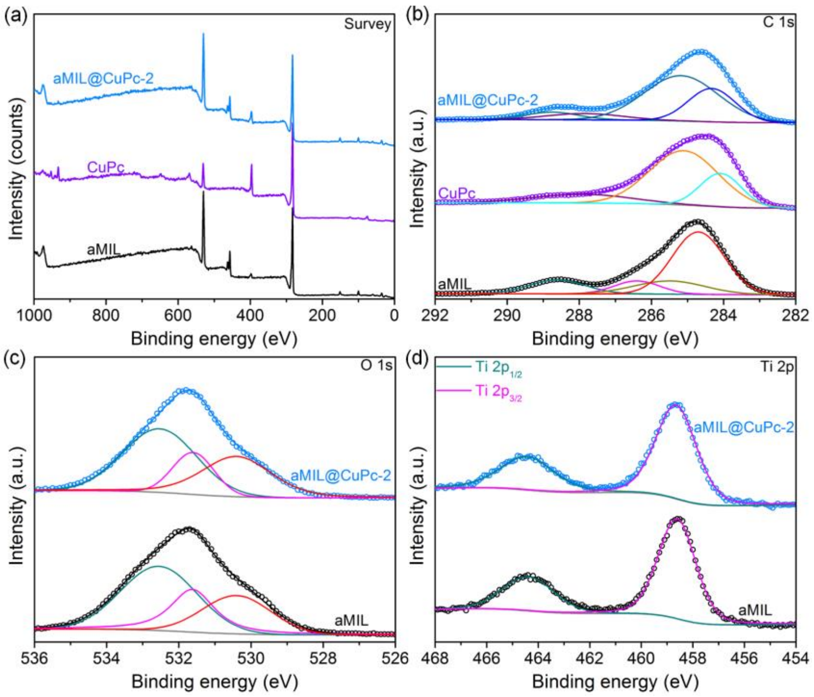

Fig. S2 a-d XPS survey spectra, and typical high-resolution XPS spectra of C 1s, O 1s and Ti 2p for aMIL, aMIL@CuPc-2 and CuPc samples.

The high-resolution XPS spectra of C 1s for aMIL and aMIL@CuPc-2 samples (Fig. S2b) exhibit peaks at 288.8, 286.4, 285.5, and 284.7 eV, which could be assigned to C=C, C–N, C–C, and C=O bonds, while the peaks located at 288.0 eV of aMIL@CuPc-2 and CuPc samples belong to N–C=N [S16, S17]. The high-resolution XPS spectra of O 1s (Fig. S2c) centered at approximately 532.6, 531.5, and 530.4 eV correspond to –OH group, C=O, and Ti–O cluster [16]. The two broad peaks located at about 464 and 459 eV in the high-resolution XPS spectra of Ti 2p (Fig. S2d) correspond to Ti^{4+} $2p_{1/2}$ and Ti^{4+} $2p_{3/2}$ [S16].

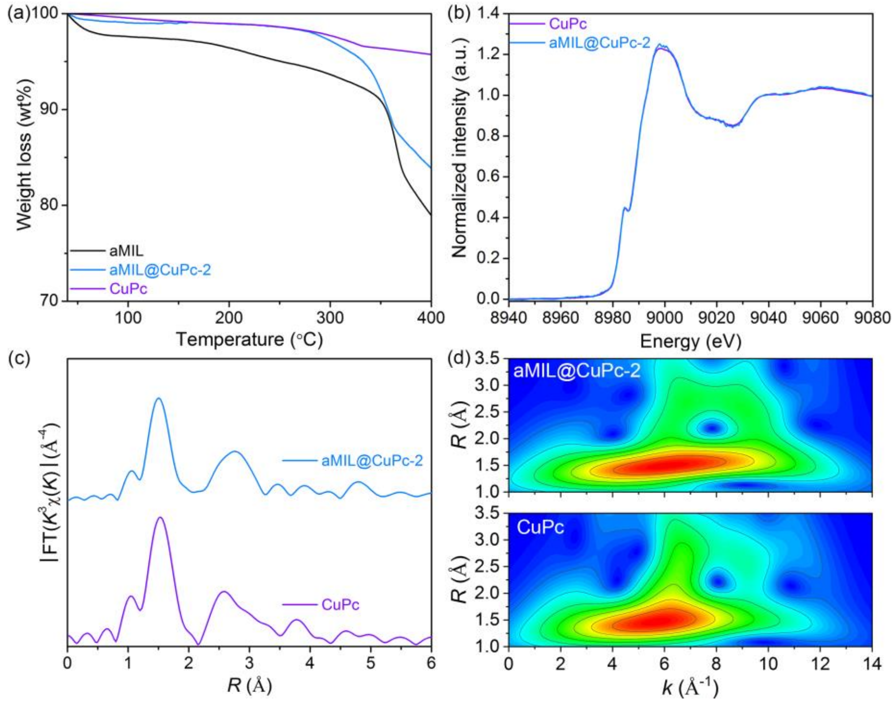

Fig. S3 a TG curves from the room temperature to 400 ºC in air of aMIL, aMIL@CuPc-2 and CuPc samples. b-d XANES, k^3-weighted FT-EXAFS and WT-EXAFS spectra at Cu K-edge of CuPc and aMIL@CuPc-2

Thermogravimetric (TG) analysis result in Fig. S3a shows that there is almost no weight loss for CuPc and aMIL@CuPc-2 samples from room temperature to ~320 ºC, which manifests CuPc molecule (β type) possesses well stability after one-pot synthesis. Cu K-edge X-ray absorption near-edge structure (XANES), k^3-weight Fourier-transformed extended X-ray absorption fine-structure (FT-EXAFS) spectra, and wavelet transform extended X-ray absorption fine-structure (WT-EXAFS) spectra of CuPc and aMIL@CuPc-2 samples (Fig. S3b-d) indicate the comparability of Cu atoms about valence states and bonding environments between CuPc and aMIL@CuPc-2 samples.

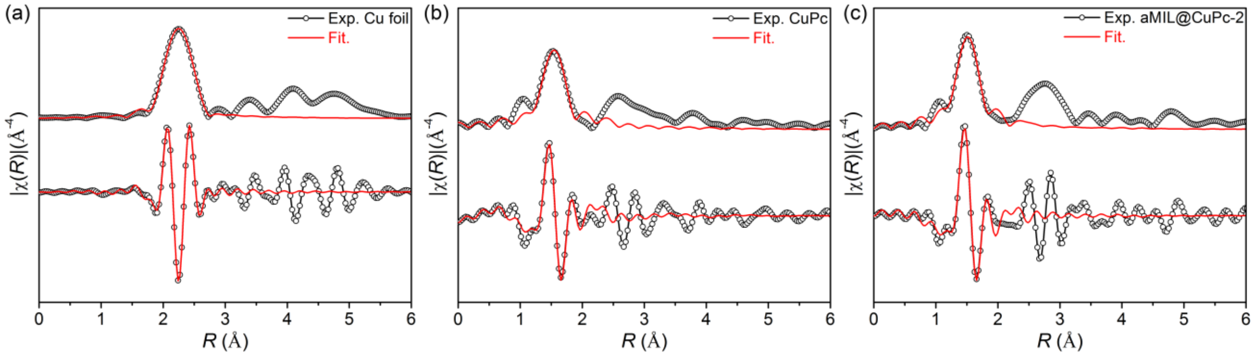

Fig. S4 a-c R space fitting spectra at Cu K-edge of Cu foil, CuPc and aMIL@CuPc-2

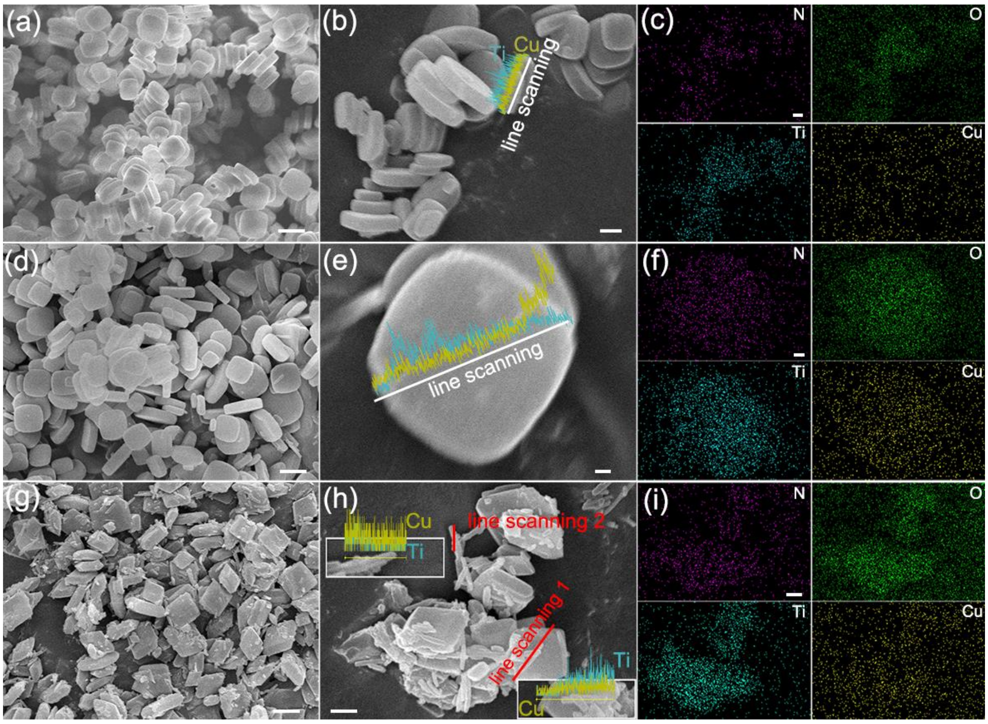

Fig. S5 **a** SEM image of aMIL. b, c SEM, EDS line scanning and mapping images of aMIL@CuPc-1. d-f SEM, EDS line scanning and mapping images of aMIL@CuPc-2. g-i SEM, EDS line scanning and mapping images of aMIL@CuPc-3. Scale bar: a, d, g 1 μm. b, c, h, i 500 nm. e, f 100 nm

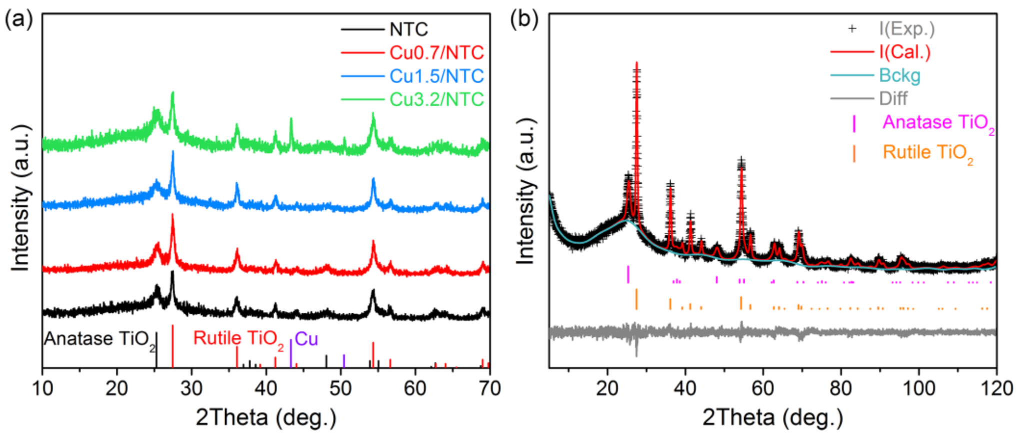

Fig. S6 a PXRD patterns of NTC, Cu0.7/NTC, Cu1.5/NTC, and Cu3.2/NTC. b Rietveld refinements curve of NTC

The PXRD patterns of derivatives shows two characteristic peaks at 25.3° and 27.4°, which could be assigned to the mixed phases of Anatase TiO_2 (JCPDS: 21-1272) and Rutile TiO_2 (JCPDS: 21-1276), respectively. To precisely illuminate the phase component of Anatase and Rutile TiO_2, the PXRD pattern of NTC sample was recorded with slower exposing times and wider angles (1 ° min^{-1}, 5° to 120°), which were carried out with GSAS software to obtain the results of Rietveld refinements (Table S2).

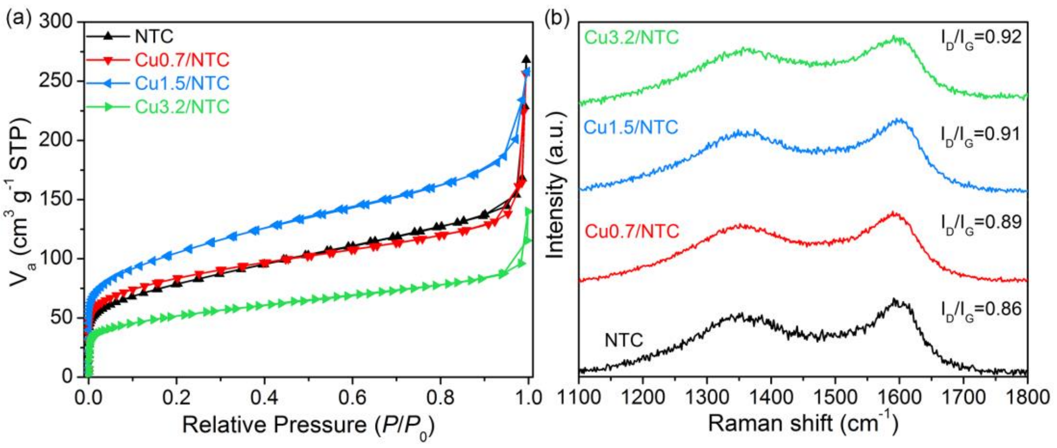

Fig. S7 a, b Nitrogen adsorption/desorption isotherms and Raman spectra of NTC, Cu0.7/NTC, Cu1.5/NTC, and Cu3.2/NTC

All isotherms (Fig. S7a) belong to reversible type I, with three stages ranging from low relative pressure (P/P_0), relative pressure ranging from 0.2 to 0.9, to near saturation pressure [S18]. Brunauer-Emmett-Teller (BET) specific surface area and pore volume (Table S3) showed that Cu1.5/NTC samples had the largest specific surface area and pore volume, which could provide more active sites. Raman spectra of NTC, Cu0.7/NTC, Cu1.5/NTC and Cu3.2/NTC samples (Fig. S7b) have D and G peaks of C at ~1352 and 1591 cm^{-1}, and the intensity ratio of D to G peak (I_D/I_G) gradually increases from 0.86 to 0.92. This indicated that the introduction of CuPc in the precursor increased the degree of graphitization of C in the derivatives and enhanced the conductivity of C materials [S19].

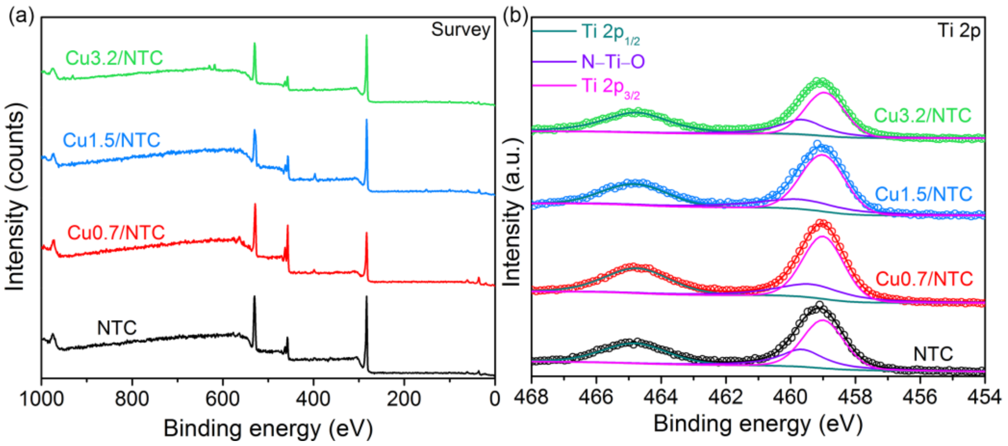

Fig. S8 a, b Survey and typical high-resolution XPS spectra of Ti 2p for NTC, Cu0.7/NTC, Cu1.5/NTC, and Cu3.2/NTC samples

XPS survey spectra of NTC, Cu0.7/NTC, Cu1.5/NTC, and Cu3.2/NTC samples (FIG. 3.12a) show that the peaks of 965-930 and 468-454 eV correspond to Cu 2p and Ti 2p, respectively. The high resolution XPS of Ti 2p (Fig. S8a) for NTC, Cu0.7/NTC, Cu1.5/NTC, and Cu3.2/NTC samples shows the peaks at 464.7 and 459 eV belonged to Ti $2p_{1/2}$ and Ti $2p_{3/2}$ of Ti^{4+}, and the peaks at 459.6 eV came from the N–Ti–O connection mode, which indicates the presence of N-doped TiO_2 [S20].

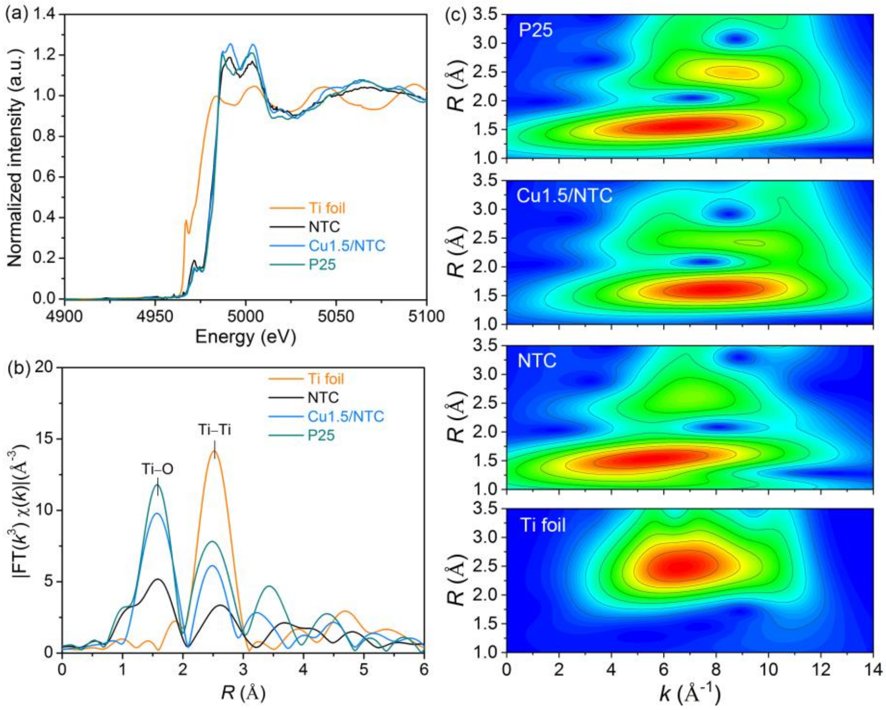

Fig. S9 a-c XANES, k^3-weighted FT-EXAFS, and WT-EXAFS at Ti K-edge of Ti foil, NTC, Cu1.5/NTC, and P25 samples

XANES spectra of NTC and Cu1.5/NTC samples at the Ti K edge (Fig. S9a) show that the Ti valence state of NTC and Cu1.5/NTC samples is +4. The peak in the first shell of P25 sample at 1.58 Å belongs to the Ti–O scattering path, while the peaks of NTC and Cu1.5/NTC samples at 1.58 and 1.57 Å correspond to Ti–O/N scattering path. WT-EXAFS spectra (FIG. 3.13c) show that the maximum WT strength of NTC (5.6 $Å^{-1}$) is smaller than that of P25 (6.3 $Å^{-1}$), indicating that there is coordination between light atoms and Ti for NTC sample (Ti–O/N). However, the maximum WT strength of Cu1.5/NTC is larger than that of NTC and P25, which could result from the strong synergistic effect of Ti–O/N and Cu species [S21].

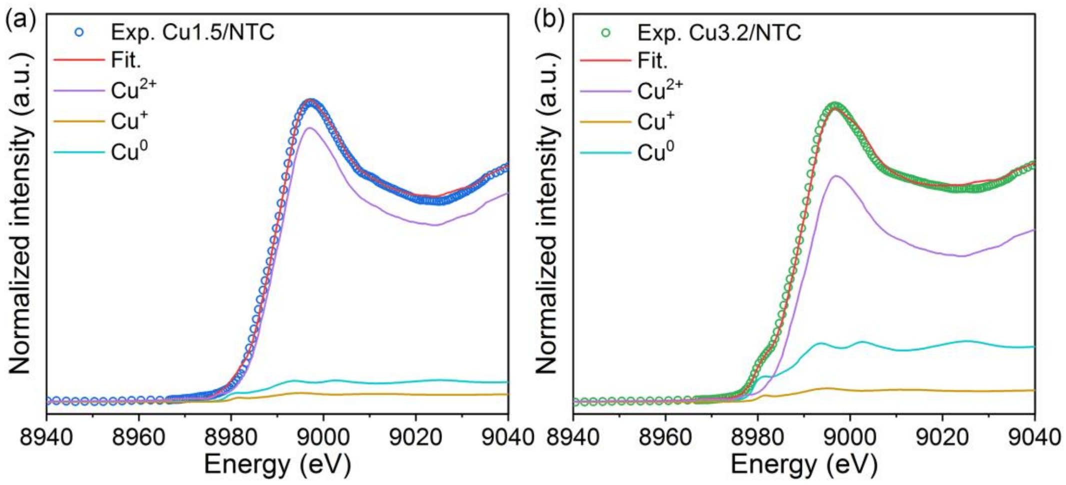

Fig. S10 a, b XANES LCF spectra of Cu1.5/NTC and Cu3.2/NTC samples

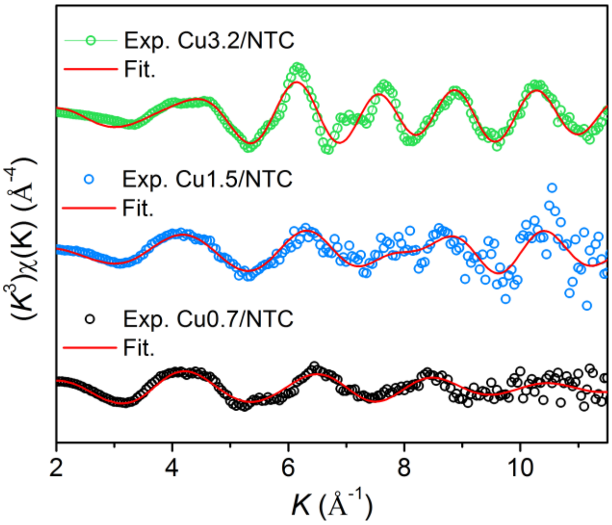

Fig. S11 The fitting spectra of k space at Cu K-edge for Cu0.7/NTC, Cu1.5/NTC, and Cu3.2/NTC

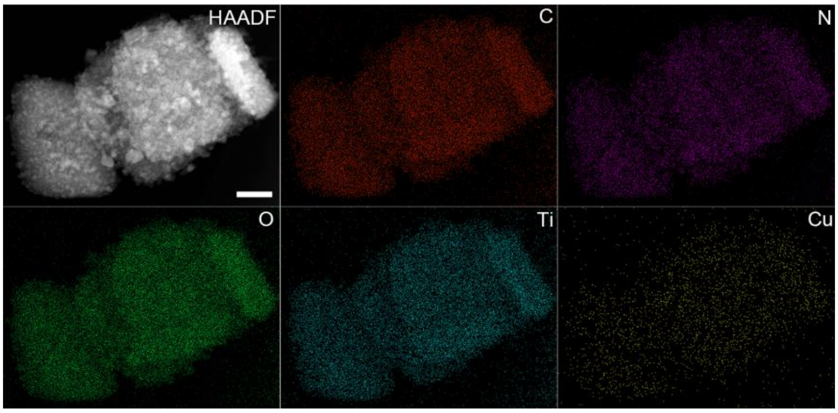

Fig. S12 EDX mapping images of Cu0.7/NTC. Scale bar: 200 nm

The Energy dispersive X-ray (EDX) element mapping image (Fig. S12) shows that elements C, N, O, Ti and Cu are uniformly distributed.

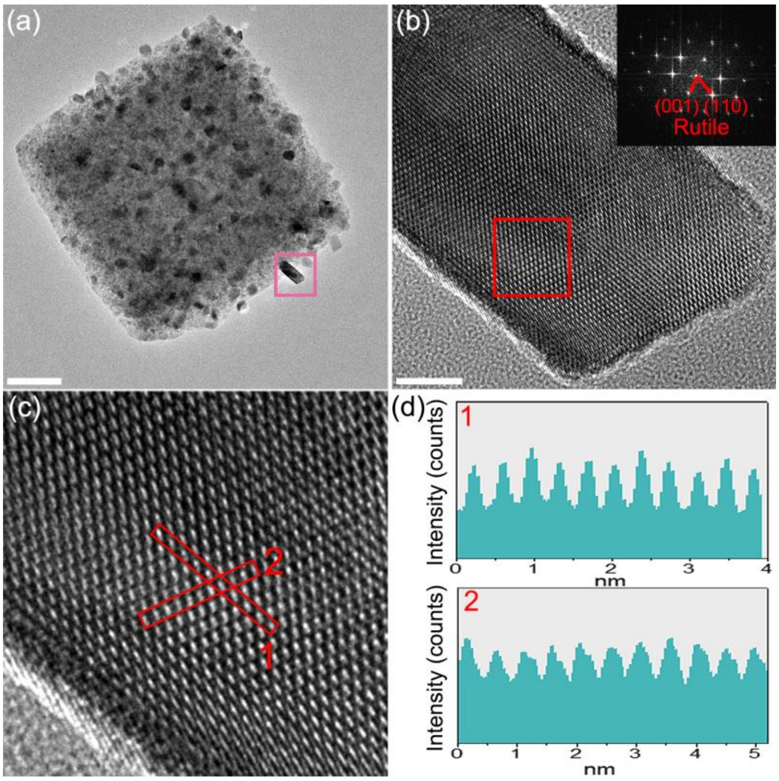

Fig. S13 HRTEM images of Cu1.5/NTC. Scale bar: a 100 nm, b 5 nm

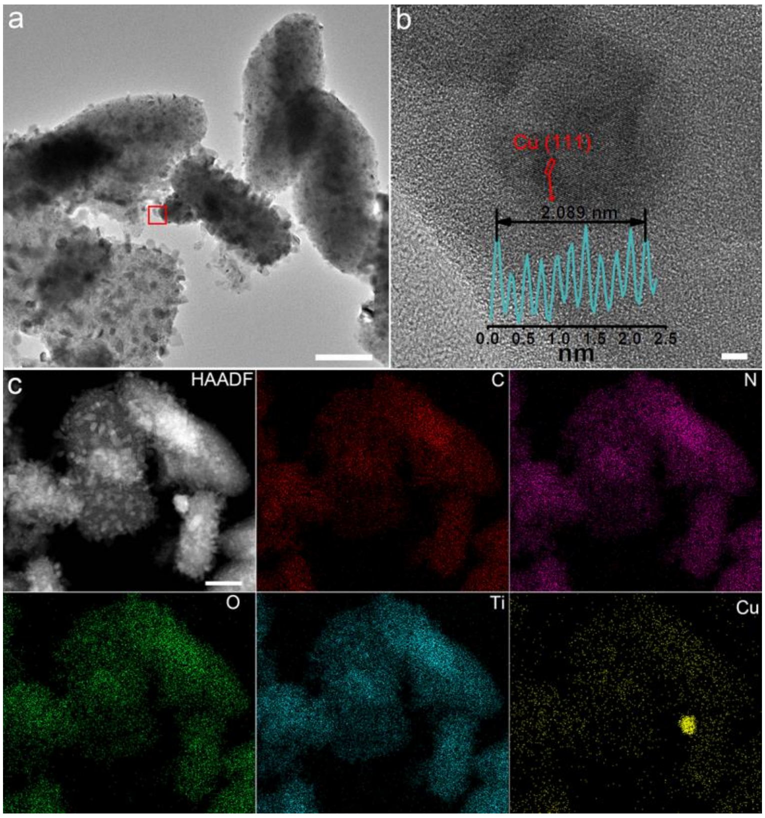

Fig. S14 a, b TEM image of Cu3.2/NTC. c EDX mapping images of Cu 3.2/NTC. Scale bar: a, c 200 nm, b 5 nm

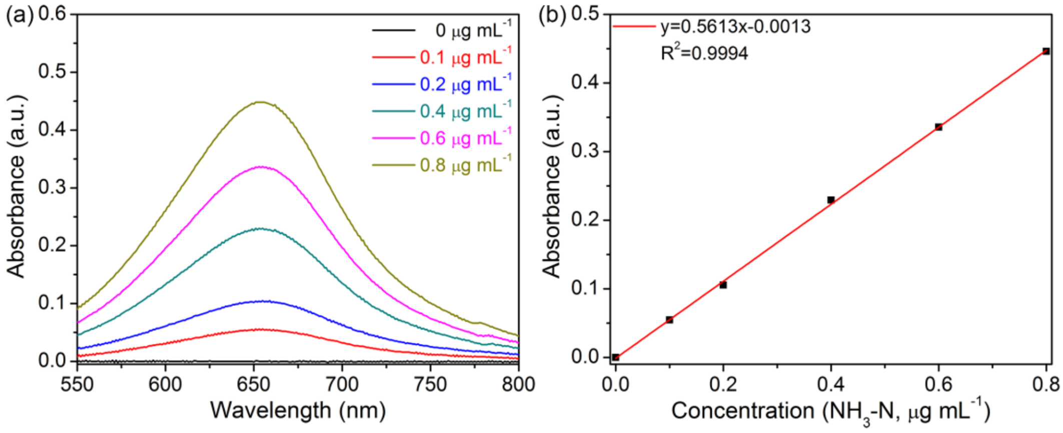

Fig. S15 a UV-Vis absorption spectra of various NH_4^+ concentrations. b Fitted calibration curve of NH_4^+

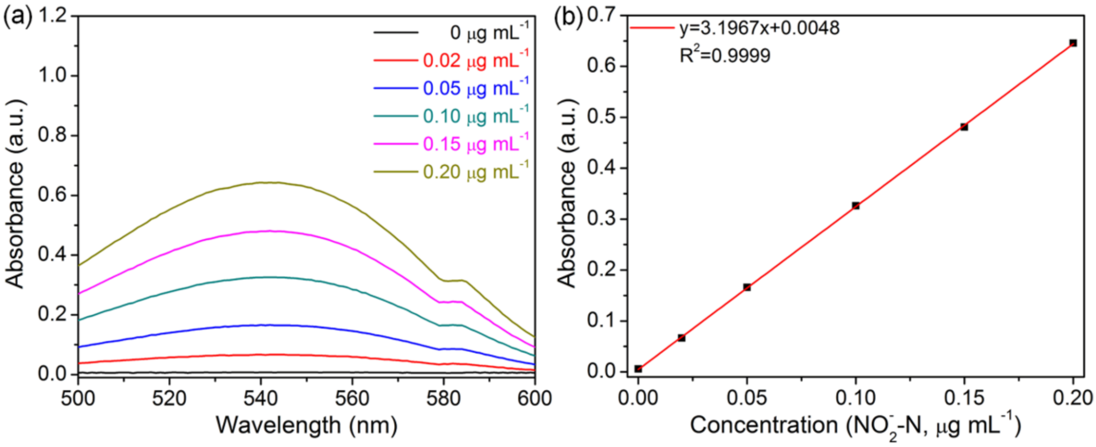

Fig. S16 a UV-Vis absorption spectra of various NO_2^- concentrations. b Fitted calibration curve of NO_2^-

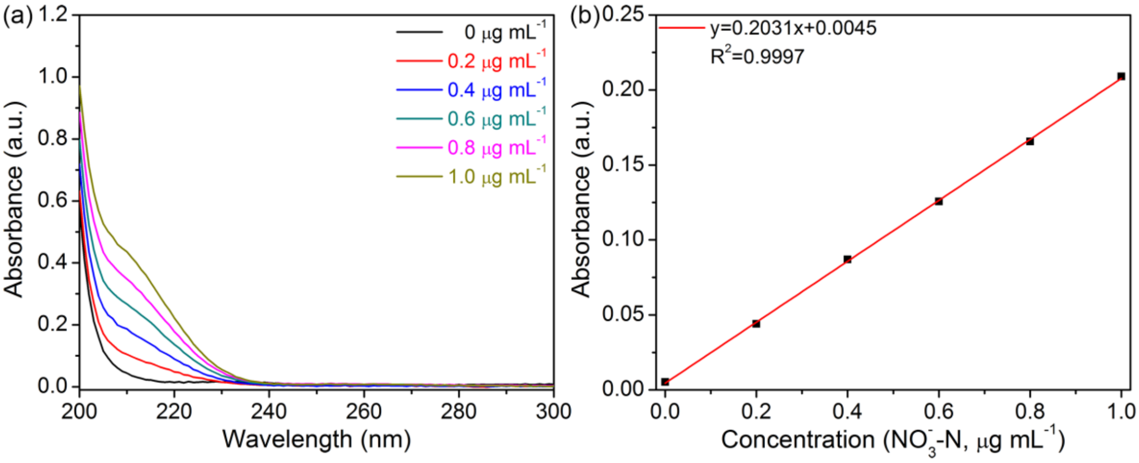

Fig. S17 a UV-Vis absorption spectra of various NO_3^- concentrations. b Fitted calibration curve of NO_3^-

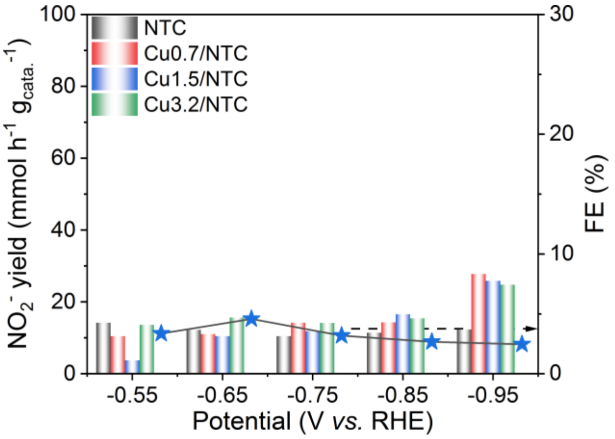

Fig. S18 Yield rate and FE of NO_2^- for NTC, Cu0.7/NTC, Cu1.5/NTC and Cu3.2/NTC samples

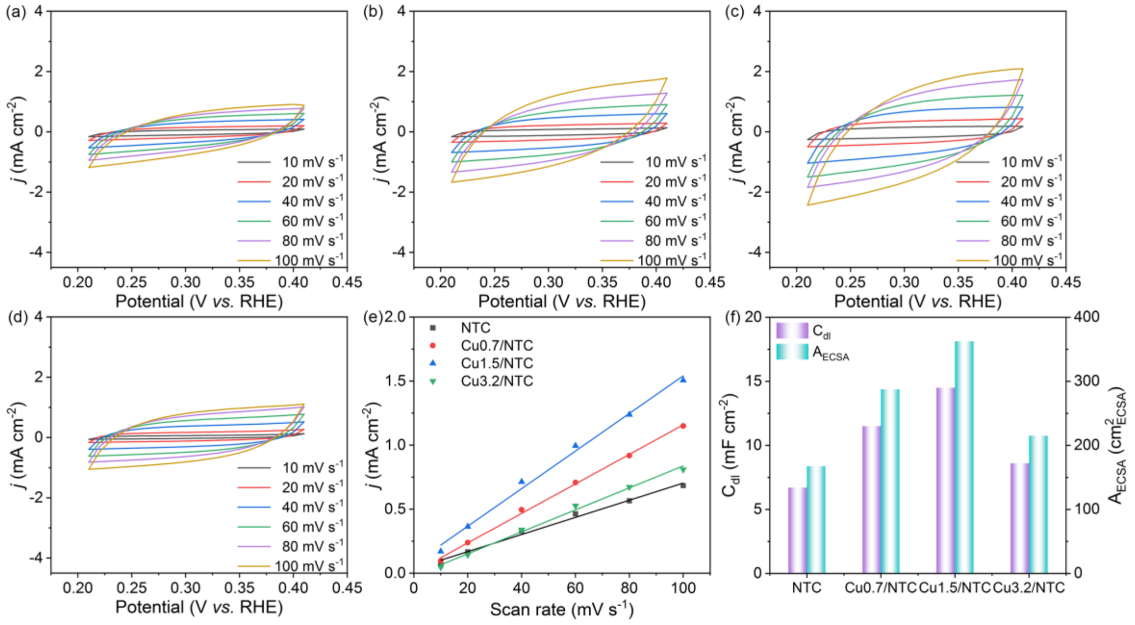

Fig. S19 a-d CV curves with different scan rates of 10, 20, 40, 60, 80, and 100 mV s^{-1} for NTC, Cu0.7/NTC, Cu1.5/NTC and Cu3.2/NTC samples. e The corresponding fitting results of current density versus scan rate. f The comparison of C_{dl} and A_{ECSA}

Cyclic voltammetry (CV) curves of NTC, Cu0.7/NTC, Cu1.5/NTC, Cu3.2/NTC electrocatalysts were measured at the sweep speed of 10, 20, 40, 60, 80, 100 mV s^{-1} (Fig. S19a-d). The corresponding curve of current density versus different CV sweep speeds and the slope (Fig. S19e) are fitted to obtain the double-layer capacitance (C_{dl}). According to Eq. (S3), the electrochemical activity specific surface area (A_{ECSA}) of NTC, Cu0.7/NTC, Cu1.5/NTC and Cu3.2/NTC are 167.5, 287.5, 362.5 and 215.0 cm^2_{ECSA} (Fig. S19f).

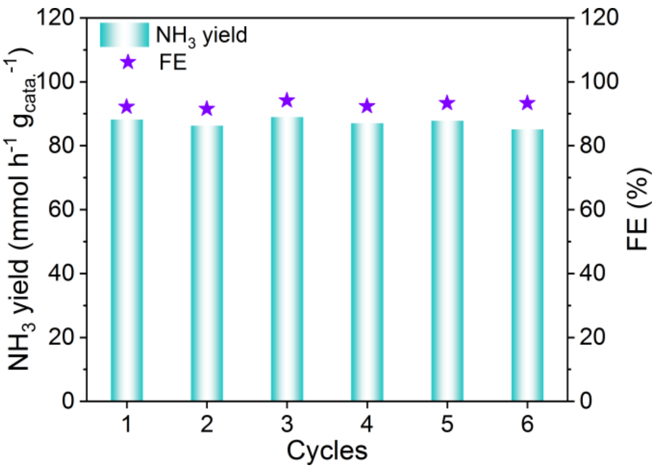

Fig. S20 Long-term experiment at –0.75 V for Cu1.5/NTC

To assess the stability of the NITRR catalyst with CuN_4&Cu_4, long-term experiment was operated with 6 cycles, and catalyst dispersed on carbon paper would be transferred to the mixed aqueous solution with Ar-saturated 0.5 M Na_2SO_4 and 50 ppm $NaNO_3$ after quickly moistening with ultrapure water.

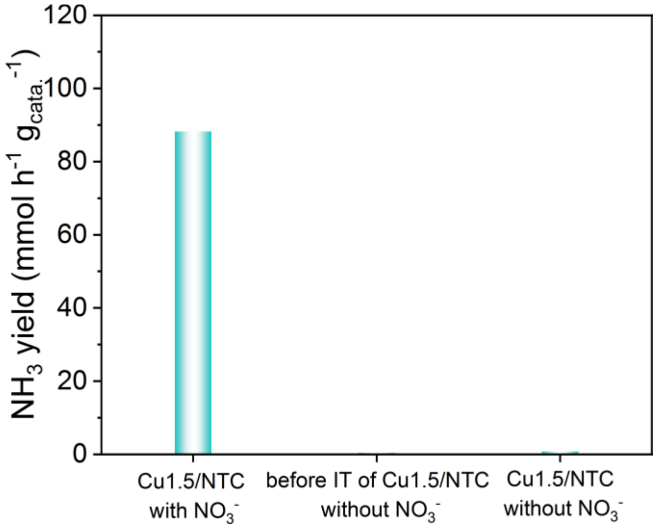

Fig. S21 NH_3 yield of Cu1.5/NTC in electrolyte with nitrate, before and after IT test without nitrate

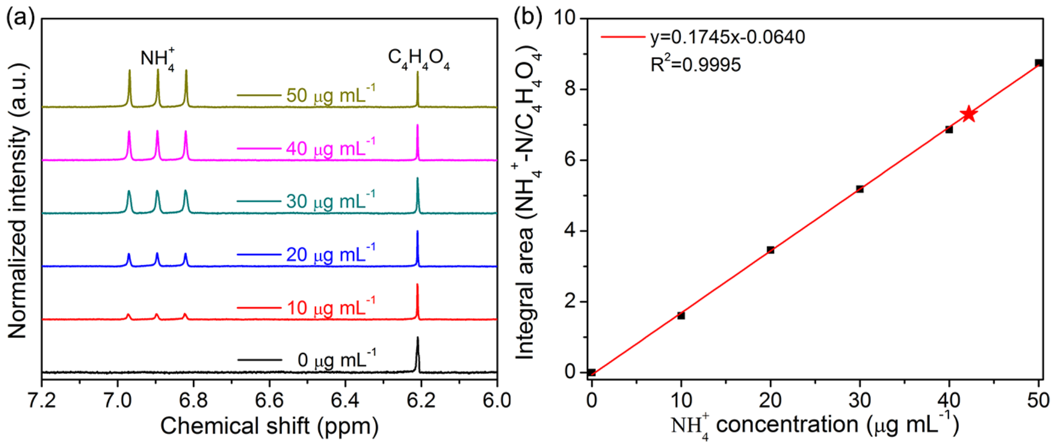

Fig. S22 a, b ^{1}H NMR spectra of $^{14}NH_4^+$-^{14}N and fitted calibration curve

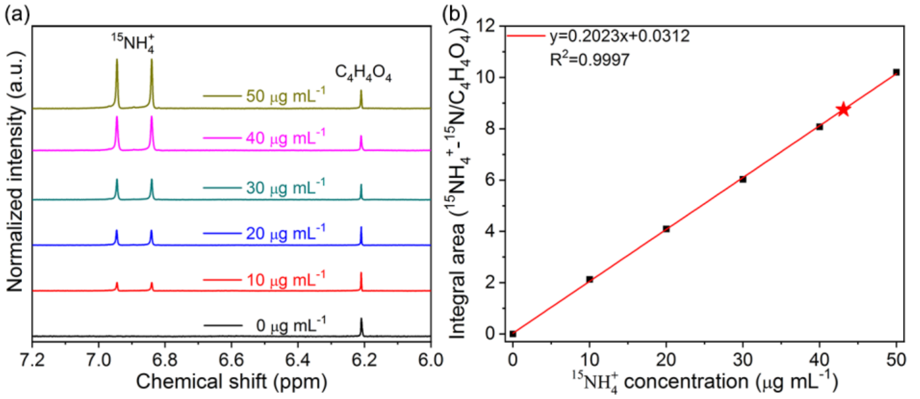

Fig. S23 a, b ^{1}H NMR spectra of $^{15}NH_4^+$-^{15}N and fitted calibration curve

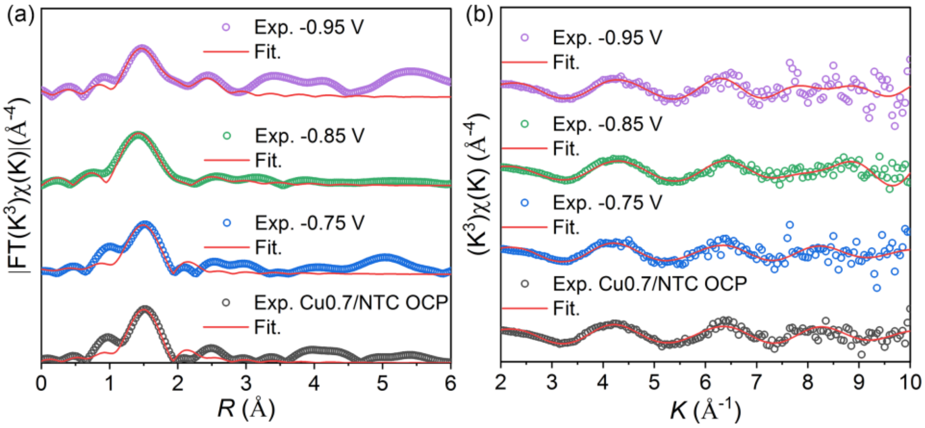

Fig. S24 a, b The fitting spectra of R and k space at Cu K-edge for Cu0.7/NTC with different potential

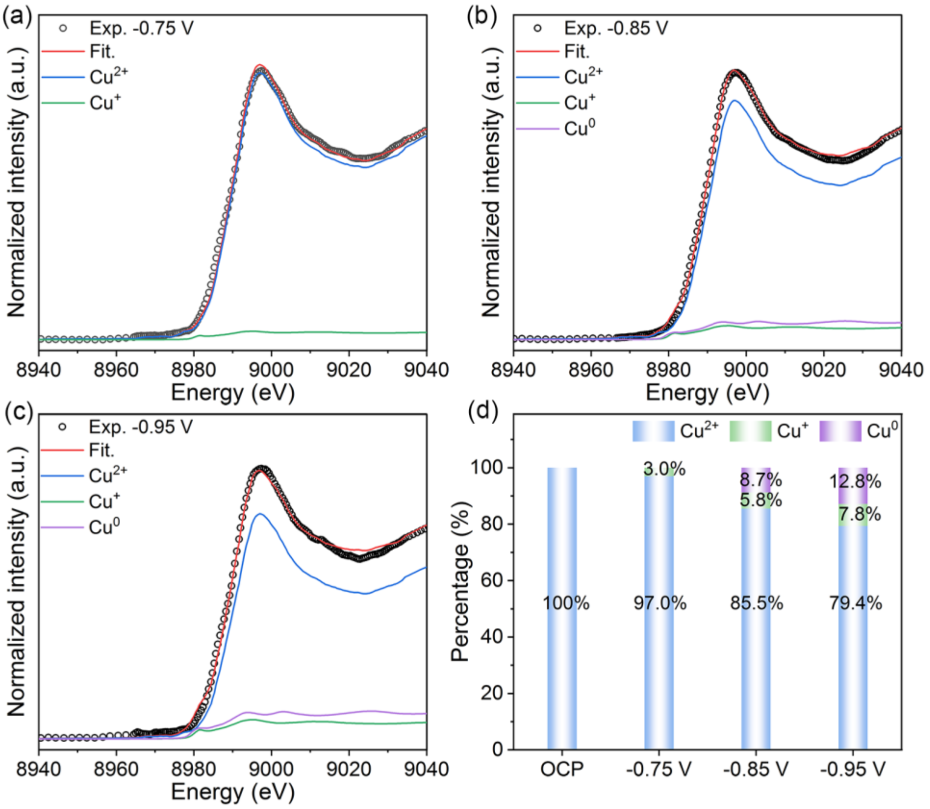

Fig. S25 a-c XANES LCF spectra of Cu0.7/NTC with different potential. d The corresponding content of Cu with different valence states

The XANES LCF spectra and the relative content of variable Cu with different valence state (Fig. S25) show that the content of Cu^{2+} decreases from 100% to 79.4% with the negative shift of reaction potential from OCP to –0.95 V, the content of Cu^{+} increases from 0 to 7.8%, while the content of Cu^{0} increases from 0 to 12.8%, which indicating that Cu0.7/NTC predominantly occur the transformation from Cu–N_4 to Cu^{+}–N_x ($x \leq 3$) at–0.75 V and reconstruction clustering behavior of Cu0.7/NTC begins at –0.85 V. The initial Cu^{2+}–N_4 configuration in Cu0.7/NTC coexists with Cu^{+}–N_x and Cu^{0} clusters along with the negative shift of reaction potential, but the Cu^{2+}–N_4 configuration is the main form of existence.

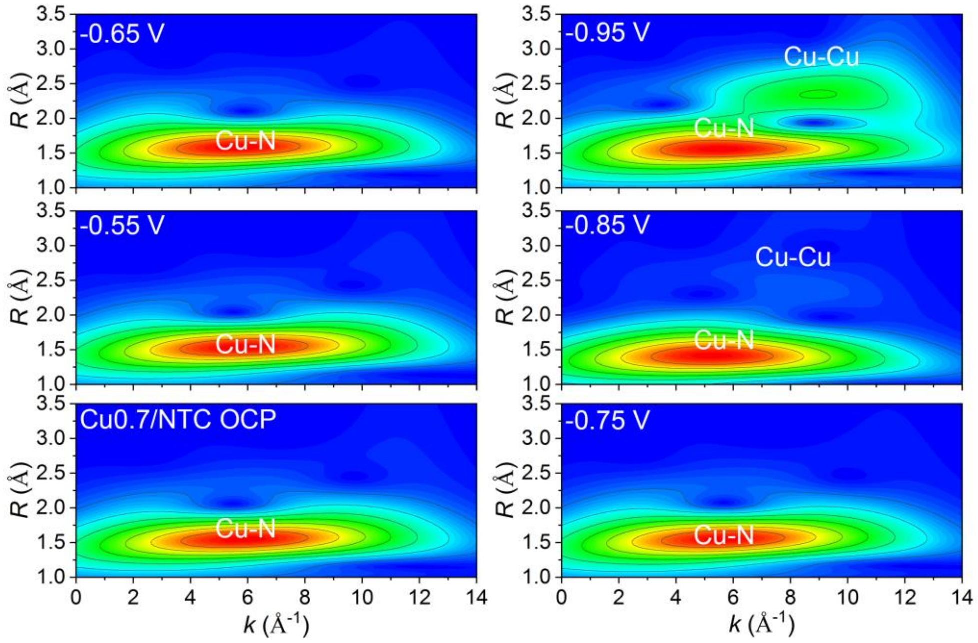

Fig. S26 WT-EXAFS at Cu K-edge for Cu0.7/NTC with different potential

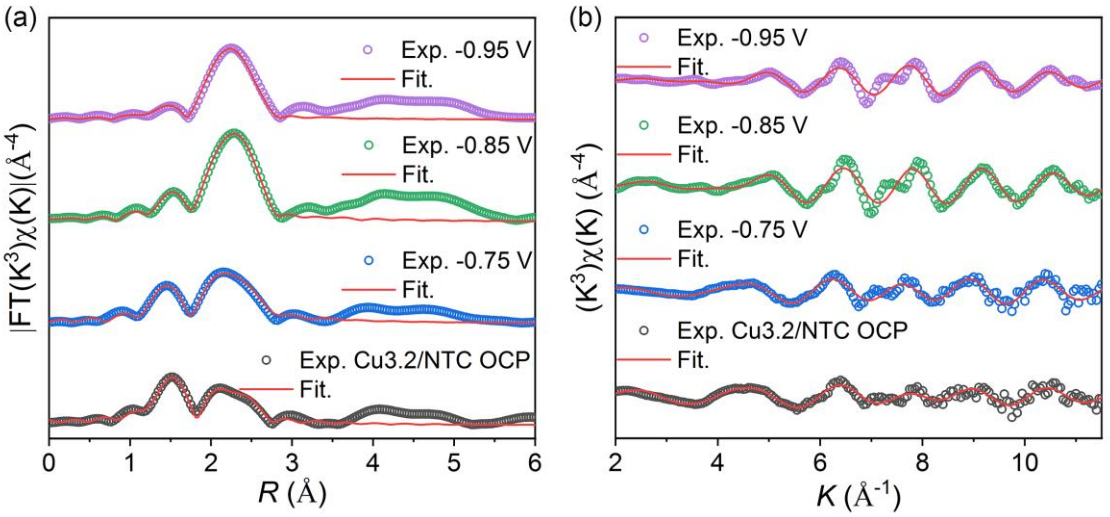

Fig. S27 a, b The fitting spectra of R and k spaces for Cu3.2/NTC with different potential

The FT-EXAFS spectra of Cu3.2/NTC at –0.95 V is resolved with Cu–N and Cu–Cu dual paths, or Cu–Cu single path, these fitting results (Table S7) reach well goodness, which indicates that Cu–N could be ignored.

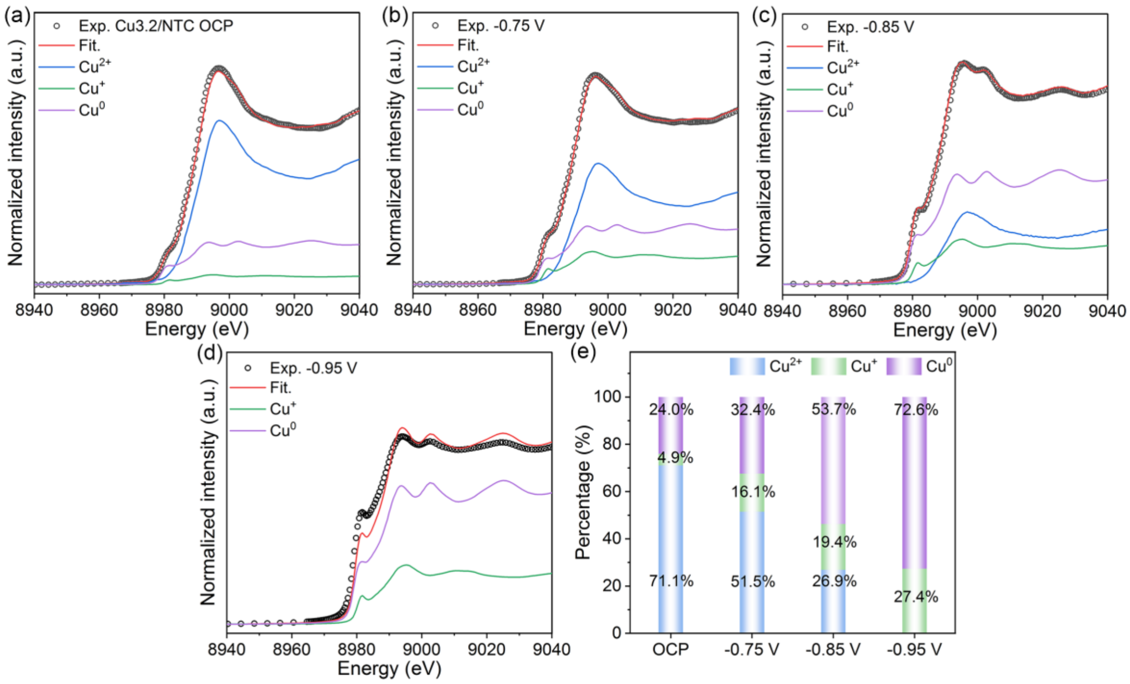

Fig. S28 a-d XANES LCF spectra of Cu3.2/NTC with different potential. e The corresponding content of Cu with different valence state

The XANES LCF spectra of Cu3.2/NTC and the relative content of variable Cu (Fig. S28) show that the content of Cu^{2+} are 74.1, 51.5 and 26.9% at OCP, –0.75 and –0.85 V, the content of Cu^{+} are 4.9, 16.1 and 19.4%, and the content of Cu^{0} are 24.0, 32.4 and 53.7%, respectively. Combining the degree of Cu clustering in the fitted spectra of FT-EXAFS and XANES LCF at –0.95 V potential for Cu1.5/NTC (Fig. 6 and S32) and Cu3.2/NTC at –0.85 V potential, we fixed the Cu–Cu bond of Cu3.2/NTC at –0.95 V potential to 12 due to the effect of X-ray on Cu NPs [S22].

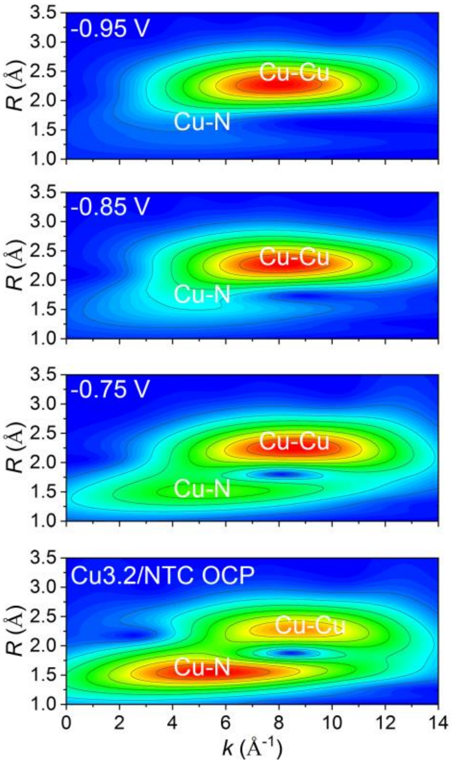

Fig. S29 WT-EXAFS at Cu K-edge for Cu3.2/NTC with different potential

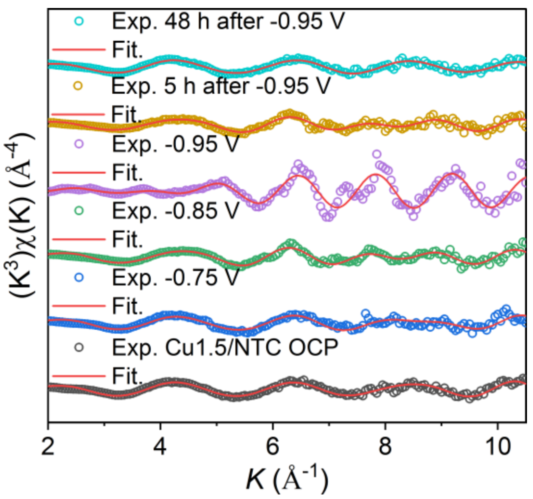

Fig. S30 The fitting spectra of k space for Cu1.5/NTC with different condition

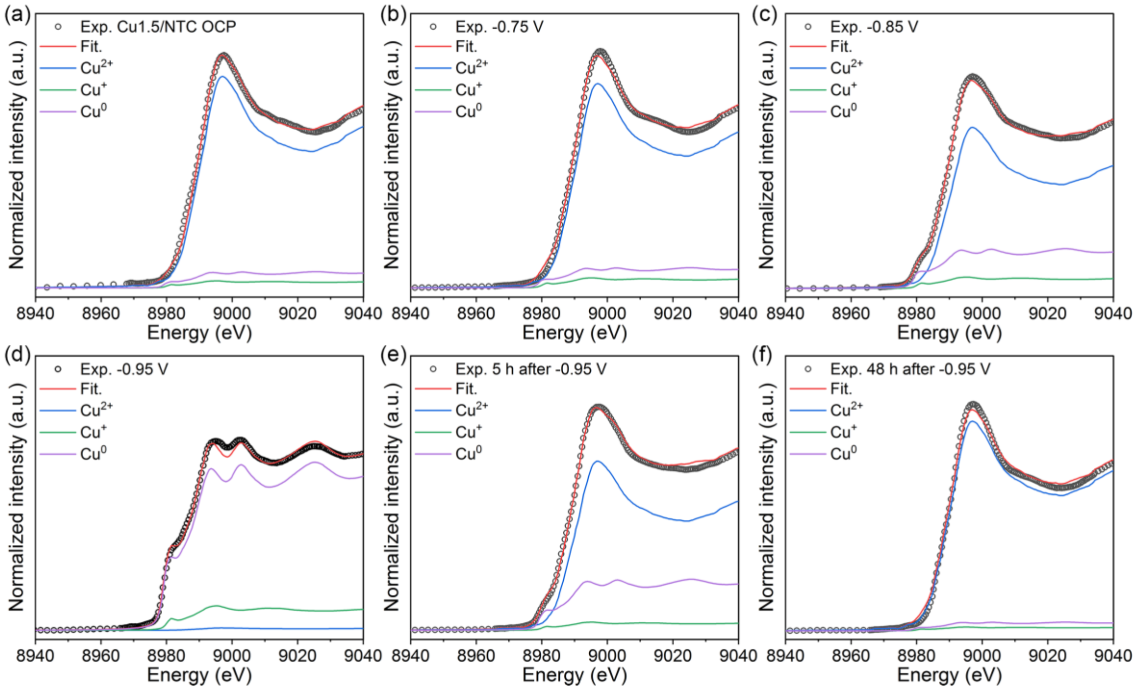

Fig. S31 a-f XANES LCF spectra of Cu1.5/NTC with different condition

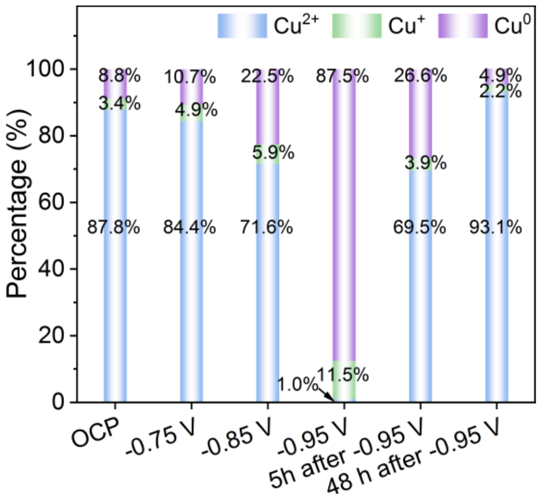

Fig. S32 The corresponding content of Cu with different valence state for Cu1.5/NTC under different condition

The XANES LCF spectra of Cu1.5/NTC and the relative content of variable Cu at OCP, –0.75, –0.85, –0.95 V, 5 and 48 h after –0.95 V (Fig. S31 and S32) show that the content of Cu^{2+} are 87.8, 84.4, 71.6, 1.0, 69.5 and 93.1%, the content of Cu^{+} are 3.4, 4.9, 5.9, 11.5, 3.9 and 2.2%, while the content of Cu^{0} are 8.8, 10.7, 22.5, 87.5, 26.6 and 4.9%, respectively. Above results uncover the non-reversible transformation for initial OCP and 5 h after –0.95 V.

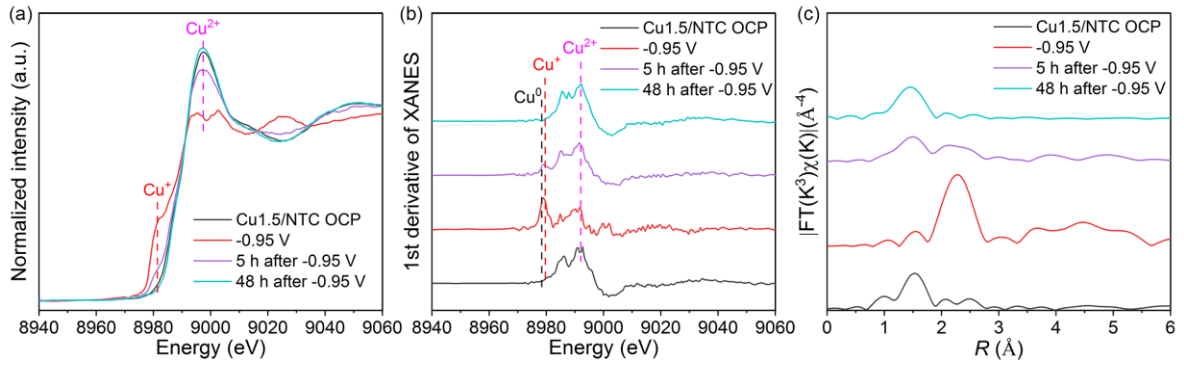

Fig. S33 a-c XANES, 1st derivative of XANES, and R space FT-EXAFS spectra of Cu1.5/NTC with the condition of OCP, –0.95 V *vs.* RHE, 5 and 48 h after –0.95 V

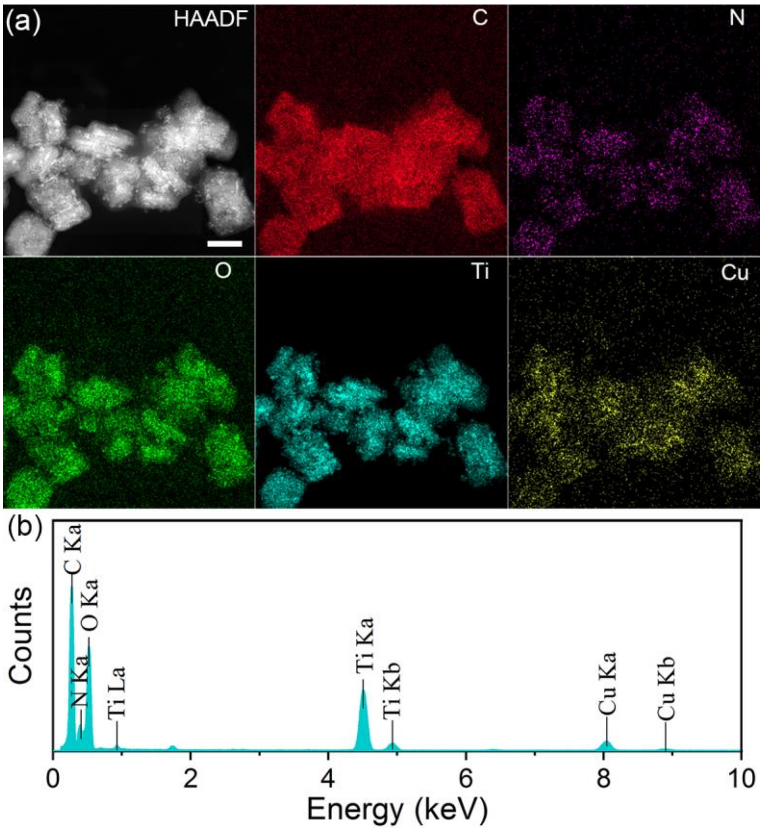

Fig. S34 a, b Ex-situ EDX mapping image and EDS spectrum of Cu1.5/NTC after –0.95 V and exposed in air. Scale bar: a 500 nm

EDX mapping and the corresponding energy dispersive spectrometer (EDS) images of Cu1.5/NTC after –0.95 V and exposed in air manifest the existence of Cu species aggregates and Cu loading is ~2.0 wt%.

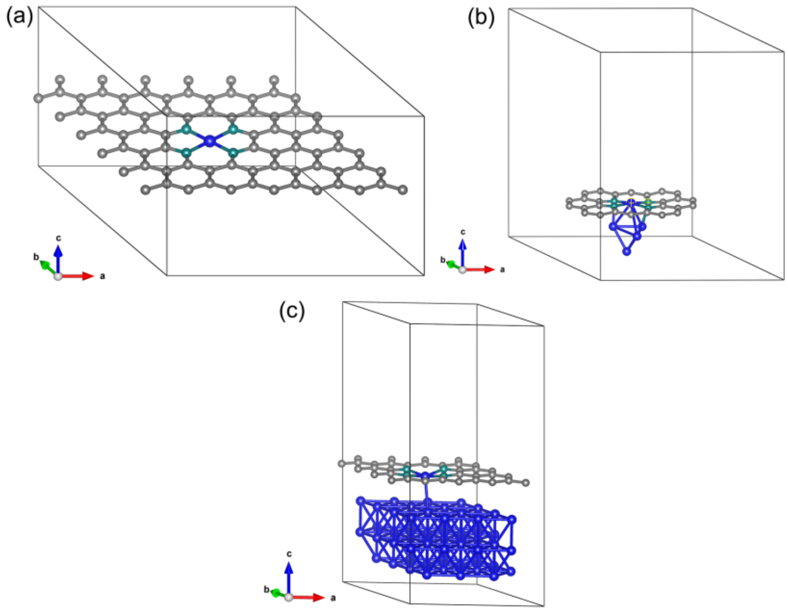

Fig. S35 a-c Calculation models of CuN_4, CuN_4&Cu_4 and CuN_4&Cu NPs. Color code: C (grey), N (green), Cu (blue)

Based on the experimental results (various Cu species, including single-atom, clusters, and nanoparticles), we build three computational models, as shown in Fig. S35. Specifically, for a single Cu atom, the CuN_4 model was adopted; for Cu cluster, the CuN_4&Cu_4 model was fabricated using the CuN_4 combined with 4 atoms Cu clusters; for Cu nanoparticles, the CuN_4&Cu NPs model was fabricated using the CuN_4 combined with facet (111) of Cu.

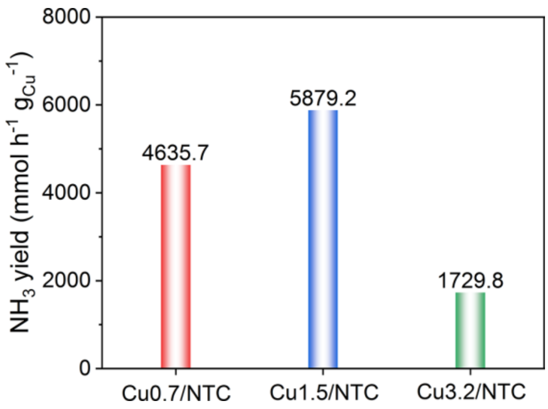

Fig. S36 Comparison of mass activity for Cu0.7/NTC, Cu1.5/NTC and Cu3.2/NTC

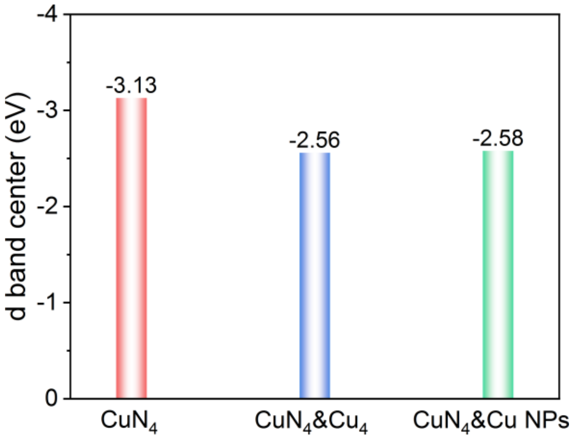

Fig. S37 *d*-band center of CuN_4, CuN_4&Cu_4 and CuN_4&Cu NPs

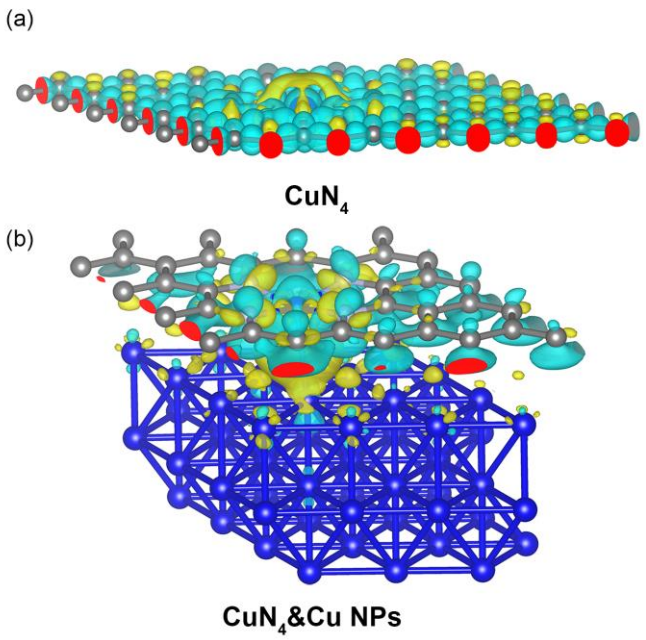

Fig. S38 EDD distribution of CuN_4 and CuN_4&Cu NPs

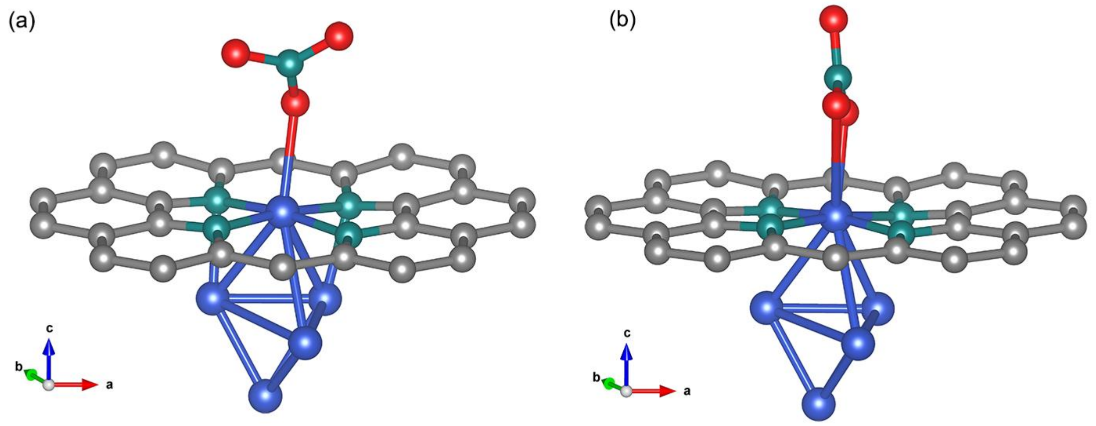

Fig. S39 a, b 1-O pattern and 2-O pattern initial configurations of NO_3^- on CuN_4&Cu_4 model

As our simulation result in Fig. S38, 2-O pattern on CuN_4&Cu_4 with –1.79 eV will be stable compared with 1-O pattern (–1.67 eV), which is consistents with previous reports [S23].

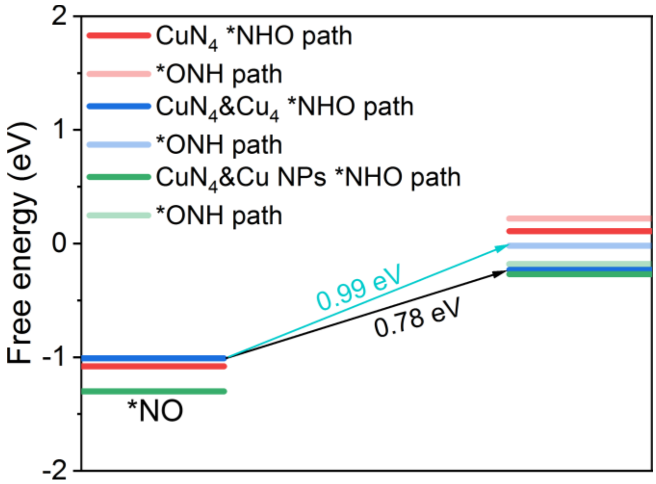

Fig. S40 The free energy of *NHO and *ONH paths for CuN_4, CuN_4&Cu_4 and CuN_4&Cu NPs

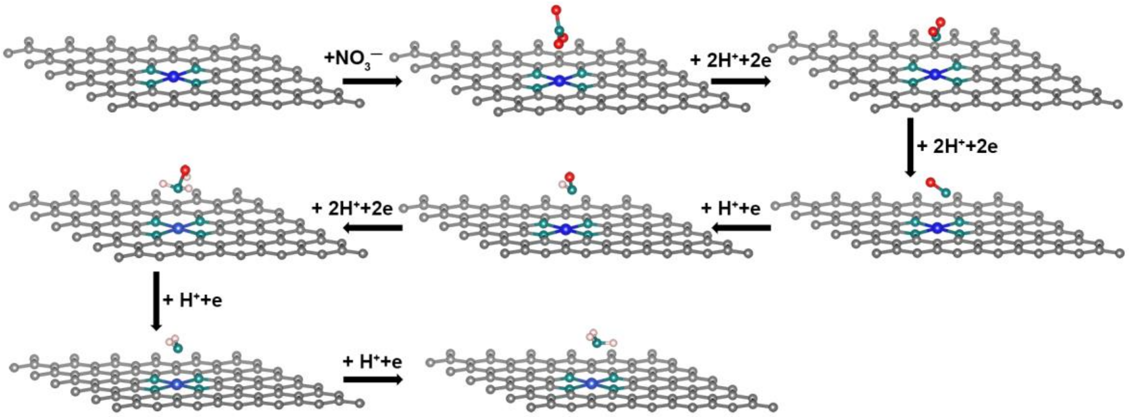

Fig. S41 The minimum energy pathway of NH_3 production for CuN_4 configuration. Color code: H (light pink), C (grey), N (green), O (red), Cu (blue)

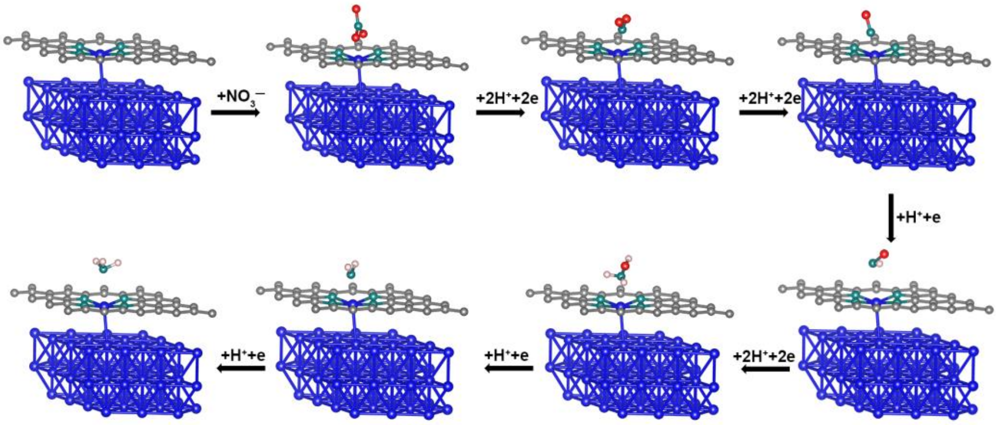

Fig. S42 The minimum energy pathway of NH_3 production for CuN_4&Cu NPs configuration. Color code: H (light pink), C (grey), N (green), O (red), Cu (blue)

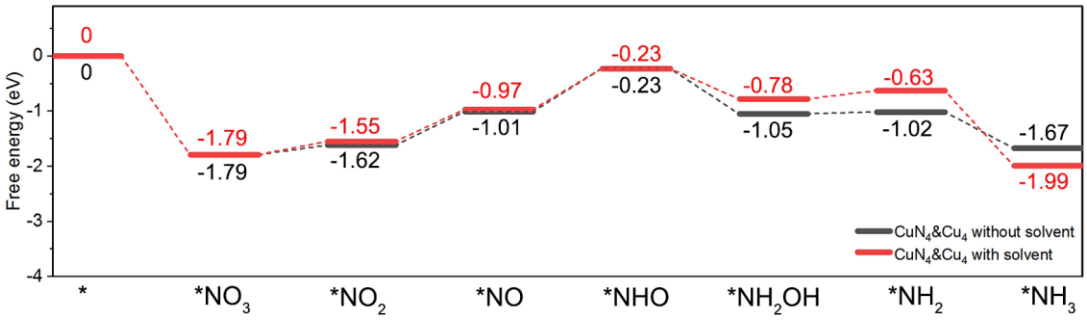

Fig. S43 The free energy paths of CuN_4&Cu_4 with and without considering the solvent effect

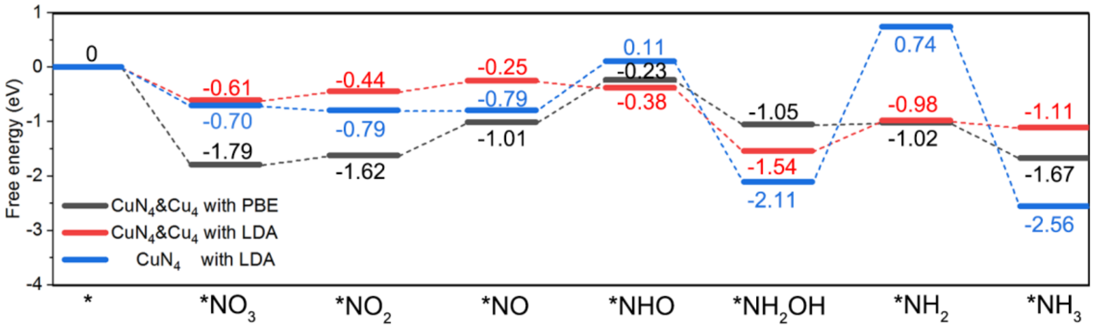

Fig. S44 The free energy paths of CuN_4&Cu_4 with PBE or LDA functional, and CuN_4 model with LDA functional

S4 Supplementary Tables

Table S1 EXAFS fitting parameters at Cu K-edge for Cu foil, CuPc, and aMIL@CuPc-2 (S_0^2=0.90)

Sample	Shell	*CN*	*R* (Å)	σ^2 (Å^2)	ΔE_0 (eV)	R factor
Cu foil	Cu–Cu	12	2.54	0.008	4.50	0.004
CuPc	Cu–N	3.72	1.95	0.002	7.47	0.015
aMIL@CuPc-2	Cu–N	4.34	1.94	0.003	4.77	0.010

CN: coordination number, ± ≤ 20%, *R*: distance between absorber and backscatter atoms, ± ≤ 20%, σ^2: Debye-Waller factor to account for both thermal and structural disorders, ± ≤ 20%, ΔE_0: inner potential correction, ± ≤ 20%, *R* factor indicates the goodness of the fit.

Table S2 PXRD Rietveld refinement parameters for NTC sample

Phases	Space group	Lattice parameter				wt%	Rwp (%)	Rwp (%)
		a = *b* (Å)	*c* (Å)	*α* = *β* = *γ* (°)	*V* (Å^3)			
Rutile TiO_2	$P4_2/mnm$	3.787(1)	9.579(8)	90	137.4(1)	29.9	6.37	8.53
Anatase TiO_2	$I4_1/amd$	4.5972(9)	2.9592(6)	90	62.54(4)	70.1		

Table S3 BET surface areas and pore volumes based on N_2 sorption results for NTC, Cu0.7/NTC, Cu1.5/NTC, and Cu3.2/NTC samples

Sample	S_{BET} (m^2 g^{-1})	Pore volume (cm^3 g^{-1})
NTC	278.5	0.3966
Cu0.7/NTC	298.2	0.4145
Cu1.5/NTC	373.1	0.3992
Cu3.2/NTC	184.1	0.2165

Table S4 EXAFS fitting parameters at Cu K-edge for Cu0.7/NTC, Cu1.5/NTC, Cu3.2/NTC (S_0^2=0.90)

Sample	Shell	*CN**	*R* (Å)	σ^2 (Å^2)	ΔE_0 (eV)	R factor
Cu0.7/NTC	Cu–N	3.99	1.94	0.007	–1.23	0.010
Cu1.5/NTC	Cu–N	4.02	1.93	0.005	4.62	0.009
	Cu–Cu	0.25/(2.84)	2.52	0.002		
Cu3.2/NTC	Cu–N	3.61	1.94	0.006	4.33	0.013
	Cu–Cu	1.78/(7.41)	2.53	0.004		

CN: coordination number, ± ≤ 20%; *R*: distance between absorber and backscatter atoms, ± ≤ 20%; σ^2: Debye-Waller factor to account for both thermal and structural disorders, ± ≤ 20%; ΔE_0: inner potential correction, ± ≤ 20%; *R* factor indicates the goodness of the fit.

Table S5 The comparison of NITRR performance to the reported Cu-based electrocatalysts

**Electrocatalysts**	**Electrolyte**	**FE (%)**	**NH_3 yeild (mmol h^{-1} $g_{cata.}^{-1}$)**	**NH_3 yeild (mmol h^{-1} cm^{-2})**	**Refs.**
O-Cu–PTCDA	0.1 M PBS+500 ppm KNO_3	85.9	2	0.026	[S24]
Cu nanosheets	0.1 M KOH+0.01 M KNO_3	99.7	2	0.01	[S25]
CuPc@MXene	0.5 M Na_2SO_4+30 ppm $NaNO_3$	94.0	80	0.04	[S26]
10Cu/TiO_{2-x}	0.5 M Na_2SO_4+200 ppm $NaNO_3$	81.3	114	0.11	[S27]
Cu/Cu-Mn_3O_4	0.5 M K_2SO_4+200 ppm KNO_3	92.4	—	0.21	[S28]
PdCu/Cu_2O	0.5 M Na_2SO_4+100 ppm$NaNO_3$	94.3	—	0.19	[S29]
i-$Cu_5Ru_1O_x$	1 M KOH+0.1 M KNO_3	94.2	—	0.48	[S30]
Cu SAC/(Cu_9)[a]	0.1 M KOH+0.1 M KNO_3	84.7	125	0.26	[S31]
Cu-UiO66 /(4 nm Cu cluster)[a]	0.5 M Na_2SO_4+0.005 M KNO_3	85.5	—	0.066	[S32]
CuO/(Cu/Cu_2O)[a]	0.5 M Na_2SO_4+200 ppm $NaNO_3$	95.8	—	0.25	[S7]
Cu1.5/NTC/(CuN_4&Cu_4)[a]	0.5 M Na_2SO_4+50 ppm $NaNO_3$	94.3	88	0.044	**This work**

[a]represents the reconstructed structure of the initial electrocatalyst at certain potential.

Table S6 EXAFS fitting parameters of Cu0.7/NTC at different potential (S_0^2=0.90)

**Potential**	**Shell**	***CN*/(*)**	***R* (Å)**	**σ^2 (Å^2)**	**ΔE_0 (eV)**	**R factor**
OCP	Cu–N	3.93	1.94	0.005	5.27	0.012
–0.75 V	Cu–N	3.97	1.95	0.006	5.67	0.014
–0.85 V	Cu–N	3.88	1.94	0.004	1.08	0.015
	Cu–Cu	0.20/(2.31)	2.53	0.001		
–0.95 V	Cu–N	3.83	1.95	0.006	6.88	0.017
	Cu–Cu	0.82/(6.43)	2.51	0.003		

CN: coordination number, ± ≤ 20%; *R*: distance between absorber and backscatter atoms, ± ≤ 20%; σ^2: Debye-Waller factor to account for both thermal and structural disorders, ± ≤ 20%; ΔE_0: inner potential correction, ± ≤ 20%; *R* factor indicates the goodness of the fit.

Table S7 EXAFS fitting parameters of Cu3.2/NTC at different potential (S_0^2=0.90)

**Potential**	**Shell**	***CN*/(*)**	***R* (Å)**	**σ^2 (Å^2)**	**ΔE_0 (eV)**	**R factor**
OCP	Cu–N	3.58	1.93	0.005	8.46	0.012
	Cu–Cu	1.79/(7.46)	2.52	0.005		
–0.75 V	Cu–N	2.89	1.93	0.005	4.11	0.002
	Cu–Cu	3.40/(10.48)	2.53	0.006		
–0.85 V	Cu–N	1.55	1.94	0.006	13.89	0.003
	Cu–Cu	6.25/(11.63)	2.55	0.008		
–0.95 V[a]	Cu–N	0.41	1.86	0.001	11.91	0.002
	Cu–Cu	5.30/(12)[c]	2.55	0.008		
–0.95 V[b]	Cu–Cu	5.08/(12)[c]	2.55	0.008	11.14	0.001

[a]Cu–N and Cu–Cu paths, [b]Cu–Cu path. *CN*: coordination number, ± ≤ 20%; *R*: distance between absorber and backscatter atoms, ± ≤ 20%; σ^2: Debye-Waller factor to account for both thermal and structural disorders, ± ≤ 20%; ΔE_0: inner potential correction, ± ≤ 20%; *R* factor indicates the goodness of the fit.

Table S8 EXAFS fitting parameters of Cu1.5/NTC with different condition (S_0^2=0.90)

**Potential**	**Shell**	***CN*/(*)**	***R* (Å)**	**σ^2 (Å^2)**	**ΔE_0 (eV)**	**R factor**
OCP	Cu–N	3.90	1.95	0.003	4.33	0.016
	Cu–Cu	0.25/(2.90)	2.53	0.003		
–0.75 V	Cu–N	3.87	1.95	0.003	5.37	0.009
	Cu–Cu	0.44/(4.12)	2.52	0.001		
–0.85 V	Cu–N	3.58	1.94	0.004	8.10	0.007
	Cu–Cu	1.64/(7.29)	2.52	0.004		
–0.95 V	Cu–N	0.59	1.93	0.008	9.64	0.005
	Cu–Cu	7.74/(8.85)	2.54	0.001		
5 h after –0.95 V	Cu–N/O	4.04	1.96	0.006	1.30	0.015

	Cu–Cu	1.72/(6.47)	2.55	0.006		
48 h after –0.95 V	Cu–N	4.06	1.94	0.003	2.23	0.016
	Cu–Cu	0.13/(2.65)	2.52	0.001		

CN: coordination number, ± ≤ 20%; *R*: distance between absorber and backscatter atoms, ± ≤ 20%; σ^2: Debye-Waller factor to account for both thermal and structural disorders, ± ≤ 20%; ΔE_0: inner potential correction, ± ≤ 20%; *R* factor indicates the goodness of the fit.

Table S9 The computed free energy (G, eV) of each elementary step during NITRR synthesis on the three models based on the PBE level

**Elementary step**	**G (CuN_4)**	**G (CuN_4&Cu_4)**	**G (CuN_4&Cu NPs)**
*	0	0	0
NO_3^-+ *→NO_3*	–1.60	–1.79	3.89
NO_3*+2H^++2e^-→NO_2*+H_2O	–1.68	–1.62	–3.04
*NO+H^++e^-→*NOH	0.11	–0.23	–0.27
NOH+2H^++e^-→NH_2OH	–1.99	–1.05	–1.16
NH_2OH+H^++e^-→NH_2+H_2O	–0.02	–1.02	–0.33
NH_2+H^++e^-→NH_3	–2.15	–1.67	–2.38

*: The adsorption site on the surface of the catalyst.

Table S10 The bond length of the key chemical bonds in reaction intermediates for CuN4, CuN_4&Cu_4, and CuN_4&Cu NPs models

**Intermediates**	**Bonds**	**Bond length (Å)**		
		**CuN_4**	**CuN_4&Cu_4**	**CuN_4&Cu NPs**
NO_3*	N–O1	1.29	1.31	1.27
	N–O2	1.26	1.25	1.25
	N–O3	1.26	1.26	1.25
*NO_2	N–O1	1.25	1.25	1.24
	N–O2	1.25	1.24	1.24
*NO	N–O	1.18	1.18	1.17
*NHO	N–O	1.24	1.24	1.23
	N–H	1.07	1.07	1.07
*NH_2OH	N–H1	1.02	1.03	1.03
	N–H2	1.03	1.03	1.03
	N–O	1.45	1.46	1.45
	O–H	0.98	0.97	0.98
*NH_2	N–H1	1.00	1.00	1.03
	N–H2	1.00	1.00	1.03
*NH_3	N–H1	1.02	1.02	1.03
	N–H2	1.02	1.02	1.03
	N–H3	1.02	1.02	1.03

Supplementary References

[S1] A.C. Larson, R.B.V. Dreele, General Structure Analysis System (GSAS). Los Alamos National Laboratory Report (LAUR) 86-748 (LANL, 2004).

[S2] B.H. Toby, EXPGUI, a graphical user interface for GSAS. J. Appl. Crystallogr. **34**, 210 (2001). https://doi.org/10.1107/S0021889801002242

[S3] B. Ravel, M Newville, ATHENA and ARTEMIS: interactive graphical data analysis using IFEFFIT. Phys. Scr. **2005**(T115), 1007 (2005). https://doi.org/10.1238/Physica.Topical.115a01007

[S4] H.P. Xu, D. Rebollar, H.Y. He, L.N. Chong, Y.Z. Liu et al., Highly selective electrocatalytic CO_2 reduction to ethanol by metallic clusters dynamically formed from atomically dispersed copper. Nat. Energy **5**, 623–632 (2020). https://doi.org/10.1038/s41560-020-0666-x

[S5] W.B. Qiu, X.Y. Xie, J.D. Qiu, W.H. Fang, R.P. Liang et al., High-performance artificial nitrogen fixation at ambient conditions using a metal-free electrocatalyst. Nat. Commun. **9**, 3485 (2018). https:// doi.org/10.1038/s41467-018-05758-5

[S6] L.C. Green, D.A. Wagner, J. Glogowski, P.L. Skipper, J.S. Wishnok et al., Analysis of nitrate, nitrite, and [^{15}N] nitrate in biological fluids. Anal. Biochem. **126**(1), 131−138 (1982). https://doi.org/10.1016/0003-2697(82)90118-X

[S7] Y.T. Wang, W. Zhou, R.R. Jia, Y.F. Yu, B. Zhang, Unveiling the activity origin of a copper-based electrocatalyst for selective nitrate reduction to ammonia. Angew. Chem. Int. Ed. **59**, 5350–5354 (2020). https://doi.org/10.1002/anie.201915992

[S8] Z.Y. Wu, M. Karamad, X. Yong, Q.Z. Huang, D.A. Cullen et al., Electrochemical ammonia synthesis via nitrate reduction on Fe single atom catalyst. Nat. Commun. **12**, 2870 (2021). https://doi.org/10.1038/s41467-021-23115-x

[S9] G. Kress, J. Furthmüller, Efficiency of ab-initio total energy calculations for metals and semiconductors using a plane-wave basis set. Comp. Mater. Sci. **6**(1), 15−50 (1996). https://doi.org/10.1016/0927-0256(96)00008-0

[S10] G. Kresse, J. Furthmüller, Efficient iterative schemes for *ab initio* total-energy calculations using a plane-wave basis set. Phys. Rev. B **54**(16), 11169 (1996). https://doi.org/10.1103/PhysRevB.54.11169

[S11] J.P. Perdew, K. Burke, M. Ernzerhof. Generalized gradient approximation made simple. Phys. Rev. Lett. **77**(18), 3865 (1996). https://doi.org/10.1103/PhysRevLett.77.3865

[S12] H.J. Monkhorst, J.D. Pack, Special points for Brillouin-zone integrations. Phys. Rev. B **13**(12), 5188 (1976). https://doi.org/10.1103/PhysRevB.13.5188

[S13] K. Mathew, R. Sundararaman, K. Letchworth-Weaver, T.A. Arias, R.G. Hennig, Implicit solvation model for density-functional study of nanocrystal surfaces and reaction pathways. J. Chem. Phys. **140**, 084106 (2014). https://doi.org/10.1063/1.4865107

[S14] K. Mathew, V.S.C. Kolluru, S. Mula, S.N. Steinmann, R.G. Hennig, Implicit self-consistent electrolyte model in plane-wave density-functional theory. J. Chem. Phys. **151**, 234101 (2019). https://doi.org/10.1063/1.5132354

[S15] W. Kohn, L.J. Sham, Self-consistent equations including exchange and correlation effects. Phys. Rev. **140**, A1133 (1965). https://doi.org/10.1103/PhysRev.140.A1133

[S16] X.N. Tan, J.L. Zhang, J.B. Shi, X.Y. Cheng, D.X. Tan et al., Fabrication of NH_2-MIL-125 nanocrystals for high performance photocatalytic oxidation. Sustain. Energ. Fuels **4**,

2823–2830 (2020). https://doi.org/10.1039/D0SE00095G

[S17] D.S. Zheng, Z.Y. Gao, X.Y. He, F.J. Zhang, L.M. Liu, Surface and interface analysis for copper phthalocyanine (CuPc) and indium-tin-oxide (ITO) using X-ray photoelectron spectroscopy (XPS). Appl. Surf. Sci. **211**(1–4), 24–30 (2003). https://doi.org/10.1016/S0169-4332(02)01333-8

[S18] M. Jiang, H.Z. Li, L.J. Zhou, R.F. Xing, J.M. Zhang, Hierarchically porous graphene/ZIF-8 hybrid aerogel: preparation, CO_2 uptake capacity, and mechanical property. ACS Appl. Mater. Interfaces **10**(1), 827−834 (2018). https://doi.org/10.1021/acsami.7b17728

[S19] D.Y. Zhai, H.D. Du, B.Y. Li, Y. Zhu, F.Y. Kang, Porous graphitic carbons prepared by combining chemical activation with catalytic graphitization. Carbon **49**(2), 725−729 (2011). https://doi.org/10.1016/j.carbon.2010.09.057

[S20] X. Bai, T. Li, Y.X. Qi, Y.X. Wang, L.W. Yin et al., One-step fabricating nitrogen-doped TiO_2 nanoparticles coated with carbon to achieve excellent high-rate lithium storage performance. Electrochim. Acta **187**, 389−396 (2016). https://doi.org/10.1016/j.electacta.2015.11.094

[S21] Y. Pan, R. Lin, Y.J. Chen, S.J. Liu, W. Zhu et al., Design of single-atom Co–N_5 catalytic site: a robust electrocatalyst for CO_2 reduction with nearly 100% CO selectivity and remarkable stability. J. Am. Chem. Soc. **140**(12), 4218–4221 (2018). https://doi.org/10.1021/jacs.8b00814

[S22] Y. Yang, I. Roh, S. Louisia, C.B. Chen, J.B. Jin et al., *Operando* resonant soft X-ray scattering studies of chemical environment and interparticle dynamics of Cu nanocatalysts for CO_2 electroreduction. J. Am. Chem. Soc. **144**(20), 8927−8931 (2022). https://doi.org/10.1021/jacs.2c03662

[S23] M.H. Jiang, Q. Zhu, X.M. Song, Y.M. Gu, P.B. Zhang et al., Batch-scale synthesis of nanoparticle-agminated three-dimensional porous Cu@Cu_2O microspheres for hghly selective electrocatalysis of nitrate to ammonia. Environ. Sci. Technol. **56**(14), 10299–10307 (2022). https://doi.org/10.1021/acs.est.2c01057

[S24] G.F. Chen, Y.F. Yuan, H.F. Jiang, S.Y. Ren, L.X. Ding et al., Electrochemical reduction of nitrate to ammonia via direct eight-electron transfer using a copper–molecular solid catalyst. Nat. Energy **5**, 605–613 (2020). https://doi.org/10.1038/s41560-020-0654-1

[S25] X.B. Fu, X.G. Zhao, X.B. Hu, K. He, Y.N. Yu et al., Alternative route for electrochemical ammonia synthesis by reduction of nitrate on copper nanosheets. Appl. Mater. Today **19**, 100620 (2020). https://doi.org/10.1016/j.apmt.2020.100620

[S26] L.X. Li, W.J. Sun, H.Y. Zhang, J.L. Wei, S.X. Wang et al., Highly efficient and selective nitrate electroreduction to ammonia catalyzed by molecular copper catalyst@$Ti_3C_2T_x$ Mxene. J. Mater. Chem. A **9**, 21771–21778 (2021). https://doi.org/10.1039/D1TA06664A

[S27] X. Zhang, C.H. Wang, Y.M. Guo, B. Zhang, Y.T. Wang et al., Cu clusters/TiO_{2-x} with abundant oxygen vacancies for enhanced electrocatalytic nitrate reduction to ammonia. J. Mater. Chem. A **10**, 6448–6453 (2022). https://doi.org/10.1039/D2TA00661H

[S28] H.J. Wang, Q.Q. Mao, T.L. Ren, T.Q. Zhou, K. Deng et al., Synergism of interfaces and defects: Cu/oxygen vacancy-rich $CuMn_3O_4$ heterostructured ultrathin nanosheet arrays for selective nitrate electroreduction to ammonia. ACS Appl. Mater. Interfaces **13**(37), 44733−44741 (2021). https://doi.org/10.1021/acsami.1c11249

[S29] H.B. Yin, Z. Chen, S.C. Xiong, J.J. Chen, C.Z. Wang et al., Alloying effect-induced

electron polarization drives nitrate electroreduction to ammonia. Chem. Catalysis **1**, 1–16 (2021). https://doi.org/10.1016/j.checat.2021.08.014

[S30] J.H. Cai, S.B. Qin, M.A. Akram, X.D. Hou, P. Jin et al., *In situ* reconstruction enhanced dual-site catalysis towards nitrate electroreduction to ammonia. J. Mater. Chem. A **10**, 12669–12678 (2022). https://doi.org/10.1039/d2ta01772e

[S31] J. Yang, H.F. Qi, A.Q. Li, X.Y. Liu, X.F. Yang et al., Potential-driven restructuring of Cu single atoms to nanoparticles for boosting the electrochemical reduction of nitrate to ammonia. J. Am. Chem. Soc. **144**(27), 12062–12071 (2022). https://doi.org/10.1021/jacs.2c02262

[S32] Y.T. Xu, M.Y. Xie, H.Q. Zhong, Y. Cao, *In situ* clustering of single-atom copper precatalysts in a metal-organic framework for efficient electrocatalytic nitrate-to-ammonia reduction. ACS Catal. **12**(14), 8698–8706 (2022). https://doi.org/10.1021/acscatal.2c02033
